# Post-translational modifications by SIRT3 de-2-hydroxyisobutyrylase activity regulate glycolysis and enable nephrogenesis

**DOI:** 10.1038/s41598-021-03039-8

**Published:** 2021-12-08

**Authors:** Luca Perico, Marina Morigi, Anna Pezzotta, Daniela Corna, Valerio Brizi, Sara Conti, Cristina Zanchi, Fabio Sangalli, Piera Trionfini, Sara Buttò, Christodoulos Xinaris, Susanna Tomasoni, Carlamaria Zoja, Giuseppe Remuzzi, Ariela Benigni, Barbara Imberti

**Affiliations:** Istituto di Ricerche Farmacologiche Mario Negri IRCCS, Centro Anna Maria Astori, Science and Technology Park Kilometro Rosso, Via Stezzano 87, 24126 Bergamo, Italy

**Keywords:** Developmental biology, Experimental organisms, Organogenesis

## Abstract

Abnormal kidney development leads to lower nephron number, predisposing to renal diseases in adulthood. In embryonic kidneys, nephron endowment is dictated by the availability of nephron progenitors, whose self-renewal and differentiation require a relatively repressed chromatin state. More recently, NAD^+^-dependent deacetylase sirtuins (SIRTs) have emerged as possible regulators that link epigenetic processes to the metabolism. Here, we discovered a novel role for the NAD^+^-dependent deacylase SIRT3 in kidney development. In the embryonic kidney, SIRT3 was highly expressed only as a short isoform, with nuclear and extra-nuclear localisation. The nuclear SIRT3 did not act as deacetylase but exerted de-2-hydroxyisobutyrylase activity on lysine residues of histone proteins. Extra-nuclear SIRT3 regulated lysine 2-hydroxyisobutyrylation (Khib) levels of phosphofructokinase (PFK) and *Sirt3* deficiency increased PFK Khib levels, inducing a glycolysis boost. This altered Khib landscape in *Sirt3*^*−/−*^ metanephroi was associated with decreased nephron progenitors, impaired nephrogenesis and a reduced number of nephrons. These data describe an unprecedented role of SIRT3 in controlling early renal development through the regulation of epigenetics and metabolic processes.

## Introduction

The nephron is the functional unit of the kidney. In mice, kidneys contain between 12,000 and 16,000 nephrons, while human kidneys are endowed with approximately 1 million nephrons on average, with significant variation between individuals^1^. Nephrogenesis is a complex process, involving multiple interacting and transitioning cell types, that begins with the invasion of the metanephric mesenchyme (MM) by the ureteric bud (UB)^2–4^. The UB grows and gives rise to the collecting duct system, while the UB tips induce the MM to condense and later epithelialise to form nephrons^2^. The activity and behaviour of renal progenitors during development dictate nephron number^5,6^. Gaining insight into the molecular mechanisms that regulate nephron endowment is of the utmost clinical interest, considering that reduced nephron number is a developmentally programmed risk for hypertension and chronic kidney diseases in adulthood^7–9^.

Several studies have shown that progenitor cell self-renewal and differentiation during kidney development are strictly dependent on epigenetic regulation. In particular, DNA methyltransferase 1 was highlighted as being indispensable for the self-renewal of nephron progenitor cells and for renal development^10,11^. In addition to DNA methylation, another repressive epigenetic marker, trimethylation of lysine 27 on histone 3 (H3K27me3), has recently been shown to be critical for renal progenitor lifespan and nephron differentiation^12,13^. Similarly, histone acetylation has been reported to regulate renal progenitor cell self-renewal and differentiation as the simultaneous deletion of both histone deacetylase 1 (*Hdac1*) and *Hdac2* in renal progenitor cells led to the arrest of nephrogenesis at the renal vesicle stage, renal hypodysplasia, and lethality shortly after birth^14^. Collectively, all these studies have shown that progenitor cell maintenance and proper differentiation require a relatively repressed chromatin landscape, considering that DNA hypomethylation^10,11^ and histone hyperacetylation^14^ have been shown to disrupt renal progenitor cell self-renewal.

More recently, the NAD^+^-dependent deacetylase sirtuins (SIRTs) have emerged as possible regulators that engage different epigenetic programmes^15–17^. SIRTs share a conserved central catalytic domain flanking the N- and C-terminal sequences, which differ between SIRTs, as they host sequences for distinct subcellular localizations, including nuclear localization signal (NLS) and mitochondrial targeting sequence (MTS)^18^. Due to their predominant nuclear localization, SIRT1, SIRT6, and SIRT7 have been found to play critical roles in epigenetics across different tissues^15–17^.

In contrast, SIRT3-5 have mostly been identified in the mitochondria, where they interact with non-histone proteins and deacetylate proteins that are involved in fatty acid oxidation, tricarboxylic acid cycle, and oxidative phosphorylation^19^. Some recent reports have also suggested that, among mitochondrial sirtuins, SIRT3 may play a role in controlling epigenetics via its deacetylase activity in the nucleus^20–22^, which is in apparent contrast with the predominant localization in the mitochondria where it regulates the major metabolic pathways^23–26^.

In humans, two different SIRT3 isoforms exist (v1, Q9NT47 and v2, Q9NT47-2). SIRT3-v1 exhibits a 142-residue amino-terminal MTS. This full-length SIRT3 is a 44 kDa protein that, following mitochondrial import, is proteolytically cleaved to generate a 28 kDa active enzyme. At variance, SIRT3-v2 results from exon skipping of the SIRT3 gene, yielding a protein that lacks the MTS^18^. In mice, two different SIRT3 isoforms have been described, which are generated by alternative splicing from three different transcript variants^27^. The long SIRT3 isoform (L-SIRT3, identifier: Q8R104-1) has a legitimate MTS, while the short SIRT3 isoform (S-SIRT3, identifier: Q8R104-2) lacks the putative amino-terminal MTS.

How different SIRT3 isoforms are expressed and how they may regulate different targets remains unclear. Further, the tissue-specific and developmental stage-specific expression of the sirtuin isoforms requires in-depth investigation. Recent evidence suggests that, particularly for SIRT1, subcellular localization and function may diverge during maturation and aging^18^. Whether different SIRT3 isoforms may control renal development through the regulation of epigenetics has never been investigated and is the aim of the present study.

Here, we report that SIRT3 is highly expressed in the early phase of kidney development and acts as a mammalian de-2-hydroxyisobutyrylase on histone lysines, an epigenetic mark associated with active gene transcription. In *Sirt3*^−/−^ mice, the lack of SIRT3 increased the lysine 2-hydroxyisobutyrylation (Khib) of H3 and H4 histones. Moreover, we found increased Khib levels of the glycolytic enzyme phosphofructokinase in metanephroi of *Sirt3* deficient mice, resulting in a boost in glycolysis. These altered Khib patterns in *Sirt3*^−/−^ mice were associated with a smaller nephron progenitor pool, delayed nephrogenesis, and reduced nephron endowment compared to wild-type counterparts. In contrast, we found that in adult mice, SIRT3 lacks any de-2-hydroxyisobutyrylase activity, while it acts as a deacetylase at the level of mitochondria.

## Methods

### Animal experiments

All procedures involving animals were performed in accordance with institutional guidelines in compliance with national (D.L.n.26, March 4, 2014), and international laws and policies (directive 2010/63/EU on the protection of animals used for scientific purposes). This study was approved by the Institutional Animal Care and Use Committees of Istituto di Ricerche Farmacologiche Mario Negri IRCCS and by the Italian Ministry of Health (approval number 16/2017-PR). This study was carried out in compliance with the ARRIVE guidelines 2.0.

The animals were randomly allocated to experimental groups. No inclusion or exclusion parameters were used in our studies. Investigators were not blinded to treatments, but no subjective assessments were made. C57BL6 × 129 wild-type (WT) and *Sirt3*^−/−^ mice (provided by Professor Frederick Alt, Harvard Medical School, Boston, MA, USA) were used. *Sirt3*^−/−^ mice were generated in a mixed genetic background, as described previously^26^. The number of animals (embryos or postnatal mice) used for each determination is indicated in the legends of the corresponding figure. The experimental unit consists in single animals for E18 and W8, whereas litter is the experimental unit for E12.5. Confounders were not controlled.

### Transmission electron microscope (TEM) analysis

Glutaraldehyde-fixed fragments of cortical kidney tissue were washed repeatedly in cacodylate buffer, post-fixed in 1% osmium tetroxide, dehydrated through ascending grades of alcohol, and embedded in Epon resin. Semithin sections were stained with toluidine blue in borax and examined using light microscopy. Thin sections (100–120 nm) were stained with uranyl acetate for morphologic analysis using a Philips Morgagni transmission electron microscope (Morgagni 268D; Philips, Brno, Czech Republic). The mitochondrial numerical density was estimated on 30 digitised TEM pictures at × 7100 for each sample and expressed as number of mitochondria per unit area (N_A_, n/µm^2^). Briefly, the mitochondrial profile area density (NA) was estimated using the ratio between the number of mitochondria and the cellular area in the image, using interactive image editing software (ImageJ; National Institutes of Health, http://rsbweb.nih.gov/ij/). Mean mitochondrial volume at W8 was measured using digitized TEM images as previously described^28^. To evaluate the extent of mitochondrial alterations, the number of mitochondria with invaginations per total number of mitochondria observed was quantified in n = 752 mitochondria for control and n = 615 for *Sirt3*^*−/−*^ mice, using interactive image editing software (ImageJ).

### Immunohistochemistry studies on kidney sections

For immunoperoxidase experiments, formalin-fixed, 3-μm paraffin-embedded kidney sections were incubated with Peroxidazed 1 (PX968H, Biocare Medical, Pacheco, CA, USA) to quench endogenous peroxidase after antigen retrieval in a decloaking chamber with DIVA (DV2004MX, Biocare Medical) to increase the reactivity of antibodies to antigens. Sections were incubated with rabbit anti-SIRT3 (ab189860, abcam, 1:100) followed by Rabbit on Rodent HRP-Polymer (RMR622G, Biocare Medical). Stainings were visualised using diaminobenzidine (BDB2004H, Biocare Medical) substrate solutions. Slides were counterstained with Mayer’s hematoxylin (MHS80-2.5 L, Bio Optica, Milan, Italy).

### In situ hybridisation

*Sirt3* mRNA in situ hybridisation (ISH) was performed on 3-µm formalin-fixed and paraffin-embedded kidney sections using the RNAscope 2.5 HD Brown Assay kit (catalog 321720, ACD Bio-techne, Minneapolis, MN, USA) according to the manufacturer’s instructions, as we previously described^29^. Specifically, the *Sirt3* target probe (300031, ACD Bio-techne, Minneapolis) used consisted of a pool of 20 double Z probes targeting the region 288–1281 of *Sirt3*, transcript variant 3, mRNA reference sequence (NM_001177804 at the National Center of Biotechnology Information) and detects all the murine *Sirt3* transcript variants.

### Protein extraction and isolation of mitochondria from renal tissue

Isolated E12.5 metanephroi were homogenised in CelLytic M (Sigma-Aldrich, C2978) supplemented with protease inhibitor cocktail (Sigma-Aldrich, P8340). Excised E18 and 8W renal tissues were homogenised in CelLytic MT (Sigma-Aldrich, C3228), supplemented with a protease inhibitor cocktail (Sigma-Aldrich, P8340). Mitochondria from E18 and W8 mouse renal tissue were isolated using the Qproteome Mitochondria Isolation Kit (Qiagen Srl., PR89801) according to the manufacturer’s protocol.

Following centrifugation at 16,000×*g* for 10 min at 4 °C, lysates were collected and total protein concentration was determined using DC™ assay (Bio-Rad Laboratories, 5000112).

### Western blot analysis

Equal amounts of proteins (30 μg) were separated on 12% SDS-PAGE under reducing conditions and transferred to nitrocellulose membranes (Bio-Rad Laboratories). In selected experiments, 2 µg of recombinant SIRT3 protein (abcam, ab97951) was used as a positive control for the identification of different SIRT3 isoforms. After blocking with 5% bovine serum albumin (BSA) in Tris-buffered saline (TBS) supplemented with 0.1% Tween-20, membranes were incubated overnight at 4 °C with 10 ml of the following antibodies: goat anti-SIRT3 (abcam, ab118334; 1:1000), rabbit anti-pan acetyl lysine (ptmbiolabs, PTM-105; 1:1000), and rabbit anti-pan 2-hydroxybutyryllysine (ptmbiolabs, PTM-801; 1:1000). Using pan 2-hydroxybutyryllysine and anti-pan acetyl lysine antibodies did not enable the identification of the specific lysine on target enzymes. Mouse anti-GAPDH (Origene Technologies, TA802519, 1:5000) or mouse anti-α-tubulin (Sigma-Aldrich, T9026; 1:2000) and mouse anti-VDAC (abcam, ab186321, 1:2000) were used as sample-loading controls in total extracts and isolated mitochondria, respectively. The signals were visualised on an Odyssey^®^FC Imaging System (LiCor) by infrared (IR) fluorescence using a secondary goat anti-rabbit IRDye 680LT antibody (LiCor, FE3680210; 1:1000) and a goat anti-mouse IRDye 800CW (LiCor, FE30926210; 1:1000) or by enhanced chemiluminescence-Western Blotting Detection Reagent (Pierce, ThermoFisher) using donkey anti-goat horseradish peroxidase (HRP)-conjugated secondary antibodies (Sigma-Aldrich, AP180P; 1:20,000). Bands were quantified through densitometry using the Image Studio Lite 5.0 (LiCor) software. All uncut gels are provided in the Supplementary Figures, where dotted red boxes indicate the portions of gel shown in the main figures of the manuscript.

### Evaluation of histone 2-hydroxyisobutyrylation and SOD2 acetylation

Equal amounts of total proteins (30 μg) were separated on 12% SDS-PAGE under reducing conditions and transferred to nitrocellulose membranes. After blocking, membranes were incubated overnight at 4 °C with rabbit anti-pan acetyl lysine (ptmbiolabs, PTM-105; 1:1000), mouse anti-histone H3 (Cell signalling, 96C10, BK3638SCST; 1,1000), mouse anti-histone H4 (Cell signalling, L64C1, BK2935SCST; 1:1000) or rabbit anti-pan acetyl lysine (Cell Signaling, BK9441, 1:1000) and sheep anti-SOD2 (Merck Millipore, 574596, 1:1000). The signals were visualised on an Odyssey^®^FC Imaging System using a secondary goat anti-rabbit IRDye 680LT antibody and a goat anti-mouse IRDye 800CW or with enhanced chemiluminescence, accordingly. Bands were quantified by densitometry using the Image Studio Lite 5.0 software. H3 and H4 2-hydroxyisobutyrylation was expressed as the ratio between the band of 2-hydroxybutyryllysine co-localising with the band corresponding to H3 and H4. SOD2 acetylation was expressed as the ratio between the band of acetylated-lysine co-localising with the band corresponding to SOD2. All uncut gels are provided in the Supplementary Figures, where dotted red boxes indicate the portions of gel shown in the main figures of the manuscript.

### Evaluation of phosphofructokinase 2-hydroxyisobutyrylation and OPA1 acetylation

Immunoprecipitation experiments were carried out using standard protocols. Briefly, 500 μg of total extracts and mitochondrial extracts were incubated overnight with 5 µl of rabbit anti-PFK antibodies (abcam, ab181064) and 5 µl of mouse anti-OPA1 antibody (BD Bioscience, 612606), respectively. After gently mixing at 4 °C overnight, 20 µl of protein A/G agarose (Santa Cruz, Santa Cruz, California, USA) were added and incubated for 60 min at 4 °C. Normal rabbit IgG (abcam, ab37415) and normal mouse IgG (Santa Cruz Biotechnology Inc., sc-2025) were used as an irrelevant isotype control. Immunoprecipitated complexes were centrifuged at 3000×g for 2 min at 4 °C and the pellet was washed 3 times with buffer containing 500 mM NaCl. Immunoprecipitated PFK and OPA1 were separated on 12% SDS-PAGE under reducing conditions and transferred to nitrocellulose membranes. After blocking, membranes were incubated overnight at 4 °C with anti-pan 2-hydroxybutyryllysine (ptmbiolabs, PTM-801; 1:1000) for PFK or rabbit anti-acetylated-lysine (Cell Signaling, BK9441, 1:1000) for OPA1. The signals were visualised on an Odyssey^®^FC Imaging System with a secondary goat anti-rabbit IRDye 680LT antibody or a secondary goat anti-mouse IRDye 800CW. Bands were quantified by densitometry using the Image Studio Lite 5.0. All uncut gels are provided in the Supplementary Figures, where dotted red boxes indicate the portions of gel shown in the main figures of the manuscript.

### Lactate dehydrogenase activity

Excised renal tissue was washed twice in normal 0.9% (w/v) sodium chloride solution and homogenised in LDH assay buffer (BioVision, K726). Total protein concentration was determined using DC™ assay (Bio-Rad Laboratories, 5000112). A lactate dehydrogenase activity assay was then performed on supernatants according to the manufacturer’s protocol at a dilution of 1:1. Measurement of OD at 450 nm was performed on the multimode microplate reader TECAN Infinite M200^®^ PRO at 37 °C at the beginning of the reaction and after 30 min of incubation.

### Phosphofructokinase activity

Excised renal tissue was washed twice in normal 0.9% (w/v) sodium chloride solution and homogenised in PFK Assay Buffer (Sigma-Aldrich, MAK093). Following centrifugation at 16,000×*g* for 10 min at 4 °C, lysates were collected and total protein concentration was determined using the DC™ assay (Bio-Rad Laboratories, 5000112). The PFK activity assay was then performed on supernatants according to the manufacturer’s protocol at a dilution of 1:1000. The measurement of OD at 450 nm was performed on the multimode microplate reader TECAN Infinite M200^®^ PRO at 37 °C.

### Immunoperoxidase analysis of nitrotyrosine

Formalin-fixed, 3-μm paraffin-embedded kidney sections were incubated with Peroxidazed 1 (Biocare Medical, Concorde, CA) to quench endogenous peroxidase, after antigen retrieval in a decloaking chamber with Rodent decloaker buffer. After blocking for 30 min with Rodent Block M (Biocare Medical), sections were incubated with rabbit anti-Nitrotyrosine antibody (Merck, 06-284, 1:100) followed by Rabbit on Rodent HRP-Polymer (Bio Optica) for 30 min at room temperature. Staining was visualized using diaminobenzidine (Biocare Medical) substrate solutions. Slides were counterstained with Mayer’s hematoxylin (Bio Optica), mounted with Eukitt mounting medium and finally observed using light microscopy (ApoTome, Axio Imager Z2, Zeiss). Negative controls were obtained by omitting the primary antibody on adjacent sections. For nitrotyrosine the score (0 = absent; 1 = faint; 2 = moderate; 3 = intense) was calculated as a weighted mean. At least 20 non-overlapping fields for each section were examined by two blinded investigators.

### Immunofluorescence analyses

Immunofluorescence analysis of whole embryonic kidneys was performed as described previously, with minor modifications^30,31^. Briefly, mouse kidneys were fixed in 10 ml of 4% paraformaldehyde (PFA) for 10 min. Kidneys were soaked in 100% cold methanol for 10 min and then incubated with mouse anti-calbindin D28k (Abcam, ab82812; 1:50), rabbit anti-SIX2 (Proteintech, 11562-1-AP; 1:50) in 50 µl of 1% bovine serum albumin (BSA) and 0.1% Tween 20 overnight at 4 °C. To detect proliferating and apoptotic cells, PFA-fixed kidneys were permeabilised with 0.1% Triton for 10 min and then incubated with phospho-histone H3 (pH3, Euroclone, BK9701S; 1:100) and Cleaved Caspase-3 (Euroclone, BK9664S; 1:50) in 0.1% Triton. Specific secondary antibodies (Jackson ImmunoResearch Laboratories) were incubated overnight at 4 °C. Nuclei were stained with DAPI (Sigma-Aldrich). In selected experiments, kidneys were labelled with Lotus Tetragonolobus (LT) lectin (Vector Laboratories, FL-1321).

Images were acquired with a Leica SP8 confocal microscope on a 40 × oil immersion using a pinhole of 1 µm for all channels and z stacks were performed at an interval of 2 µm resulting in non-overlapping images. Positive cells were quantified in each z stack image using the Orbit image analysis software (Actelion Pharmaceuticals Ltd). In whole metanephroi, SIX2-positive cells were quantified per nephrogenic niche, which is defined as reported previously^32^. Proliferating or apoptotic cells were quantified in z stack optical sections in the niche or in UB and expressed as percentage of positive cells on total cell (DAPI-positive cells).

The thickness of the nephrogenic area, identified by the presence of SIX2-positive cells, was quantified on renal sections of WT and *Sirt3*^−/−^ mice on E18. Sections of 3 µm from PFA-fixed kidneys were stained for rabbit anti-SIX2 (Proteintech, 1:50), wheat germ agglutinin lectin (WGA, Vector Laboratories; 1:400) and DAPI. Images were acquired with a 63 × objective. The area occupied by SIX2-positive cells was identified by drawing a line following the distribution of SIX2-positive cells within the inner part of the tissue. The distance from the edge was measured in at least 10 different points per section in n = 3 WT and n = 4 *Sirt3*^*−/−*^ mice.

### Organ culture experiments

Embryonic kidneys were cultured as previously described, with minor modifications^30,31^. Briefly, embryonic day (E) 11.5 mouse kidneys were isolated and placed on a 5-μm polycarbonate filter (Merck Millipore Ltd.) supported by a metal grid at the air-medium interface. Kidneys were cultured in Advanced DMEM (Invitrogen, 12494) supplemented with 2% Embryonic Stem Cell Fetal Bovine Serum (ES-FBS, Invitrogen, 16141079), 1% L-glutamine (Invitrogen, 25030024) and 1% penicillin/streptomycin (Invitrogen, 15140122).

### *Sirt3* silencing, overexpression and tubulogenesis assay in mIMCD3

Murine inner medullary collecting duct (mIMCD3) cells were cultured as previously described^33^. mIMCD3 seeded on 9.6-cm^2^ tissue culture plates at 70% confluence were transfected with 50 nM Silencer Select predesigned siRNA mouse *Sirt3* (Assay ID: s82219, Life Technologies) or with control nontarget siRNA (Silencer Select Negative Control #2siRNA, si*Null*) using Lipofectamine 2000 reagent (Invitrogen), according to the manufacturer’s protocol.

For *Sirt3* overexpression, we designed a mammalian gene expression vector containing the short isoform of codon optimized mouse *Sirt3* under the CMV promoter (pRP[Exp]-EGFP-CMV > {mSirt3[NM_022433.2]*(co)}, VectorBuilder). The siRNA recognition sequence of the codon optimized cDNA differs from the endogenous one (NM_022433.2) by six base pairs, thus preventing its recognition by *Sirt3* siRNA. Briefly, mIMCD3 cells seeded on 9.6-cm^2^ tissue culture plates at 70% confluence were transfected with 2.5 μg plasmid DNA using Lipofectamine 2000 reagent according to the manufacturer’s instructions. Twenty-four hours after transfection, exogenous *Sirt3* expression was analyzed using Real Time PCR. For rescue experiments, cells were transfected with 2.5 μg plasmid DNA in the presence of 50 nM *Sirt3* siRNA or with si*Null*.

For the tubulogenesis assay, 24 h after *Sirt3* silencing or overexpression, mIMCD3 cells were resuspended in 2.4 mg/ml rat-tail collagen type I (Corning-Costar, 354236) and seeded into polyester Transwell membrane inserts (0.4 μm pore size; Corning-Costar, CC3460) at a concentration of 5 × 10^2^ cells/μl collagen. After collagen polymerization (45 min at 37 °C), 1 ml of culture medium [Advanced DMEM (Cat#12491023, ThermoFisher) enriched with 2% FBS (Cat#10082147, ThermoFisher) and 1% L-glutamine (Cat#25030024, ThermoFisher)] supplemented with 1 μg/ml heparin (VERACER, Medic Italia S.r.l., Roma, Italy), 40 ng/ml hepatocyte growth factor (HGF; Cat#100-39, PeproTech), 100 ng/ml glial-derived neurotrophic factor (GDNF; Cat#ab73450, Abcam), 200 ng/ml fibroblast growth factor-1 (FGF1; Cat#100-17A, PeproTech) and 100 ng/ml fibroblast growth factor-7 (FGF7; Cat#100-19, PeproTech) was added to the bottom of each well. Cells were incubated in standard conditions (37 °C, 5% CO_2_, 20% O_2_, humidified atmosphere) and cultures were monitored by light microscopy for 3 days. Culture medium was changed 2 days after seeding. The overall mIMCD3 cell-derived tubules were quantified in bright-field images of the cultures. Data are expressed as percentage of ramified tubules over total tubules.

In selected experiments, mIMCD3 cells were sonicated in CelLytic MT (Sigma-Aldrich, C3228) supplemented with protease inhibitor cocktail (Sigma-Aldrich, P8340) or in PFK Assay Buffer (Sigma-Aldrich, MAK093) for the evaluation of phosphofructokinase 2-hydroxyisobutyrylation or phosphofructokinase activity.

### Real-time PCR of endogenous and exogenous *Sirt3*

The evaluation of *Sirt3* expression was performed using real time PCR 24 h after transfection. Briefly, mIMCD3 were harvested in TRIzol reagent (Invitrogen, Life Technologies), and total RNA was extracted according to the manufacturer’s instructions. Contaminating genomic DNA was removed using RNase-free DNase (Promega) for 1 h at 37 °C. The first-strand cDNA (2.5 μg) was produced using a SuperScript VILO cDNA Synthesis Kit (Life Technologies), according to the manufacturer’s procedure. No enzyme was added for reverse transcriptase–negative controls (RT^−^). To amplify the cDNA of endogenous and exogenous *Sirt3*, SYBR Green PCR Master Mix and the following primers (300 nM) were used: mSirt3 forward 5′-CAGCTTGTCTGAAGCAGTAC-3′, Reverse 5′-CCACACCATGAACTACATCC-3′; Exogenous mSirt3 forward 5′-TCCCCGACTTTAGAAGCCCT-3′, reverse 5′-AGCTCCTTGGCCAGCATAAA-3′; glyceraldehyde 3-phosphate dehydrogenase forward 5′-TCATCCCTGCATCCACTGGT-3′ reverse 5′-CTGGGATGACCTTGCCCAC-3′. cDNA content was calculated using the 2^−ΔΔCt^ technique in each sample, using the cDNA expression in untreated mIMCD3 as calibrator.

### Renal histology and estimation of number of glomeruli

For renal histology, Duboscq-Brazil-fixed, 3 µm paraffin-embedded kidney sections were stained with hematoxylin/eosin. Maceration of whole kidneys was performed with HCl according to MacKay et al.^34^. Then, 500 μl of macerate was pipetted into a cell culture dish with a grid and glomeruli per area were counted.

### Statistics

Results are expressed as means ± s.e.m. Data analysis was performed with Prism Software (GraphPad Software Inc.). Comparisons were made using unpaired Student’s *t*-test or ANOVA corrected with Bonferroni coefficient, as appropriate. Levene’s test was used to assess homogeneity of variance between the groups. Statistical significance was defined as *P* < *0.05*. All representative images were obtained from independently repeated experiments. Image analyses were performed in a blinded fashion. Investigators were unaware of the genotype of the animals or the identity of samples during assessment.

## Results

### SIRT3 is highly expressed in the embryonic kidney

**Figure 1:**
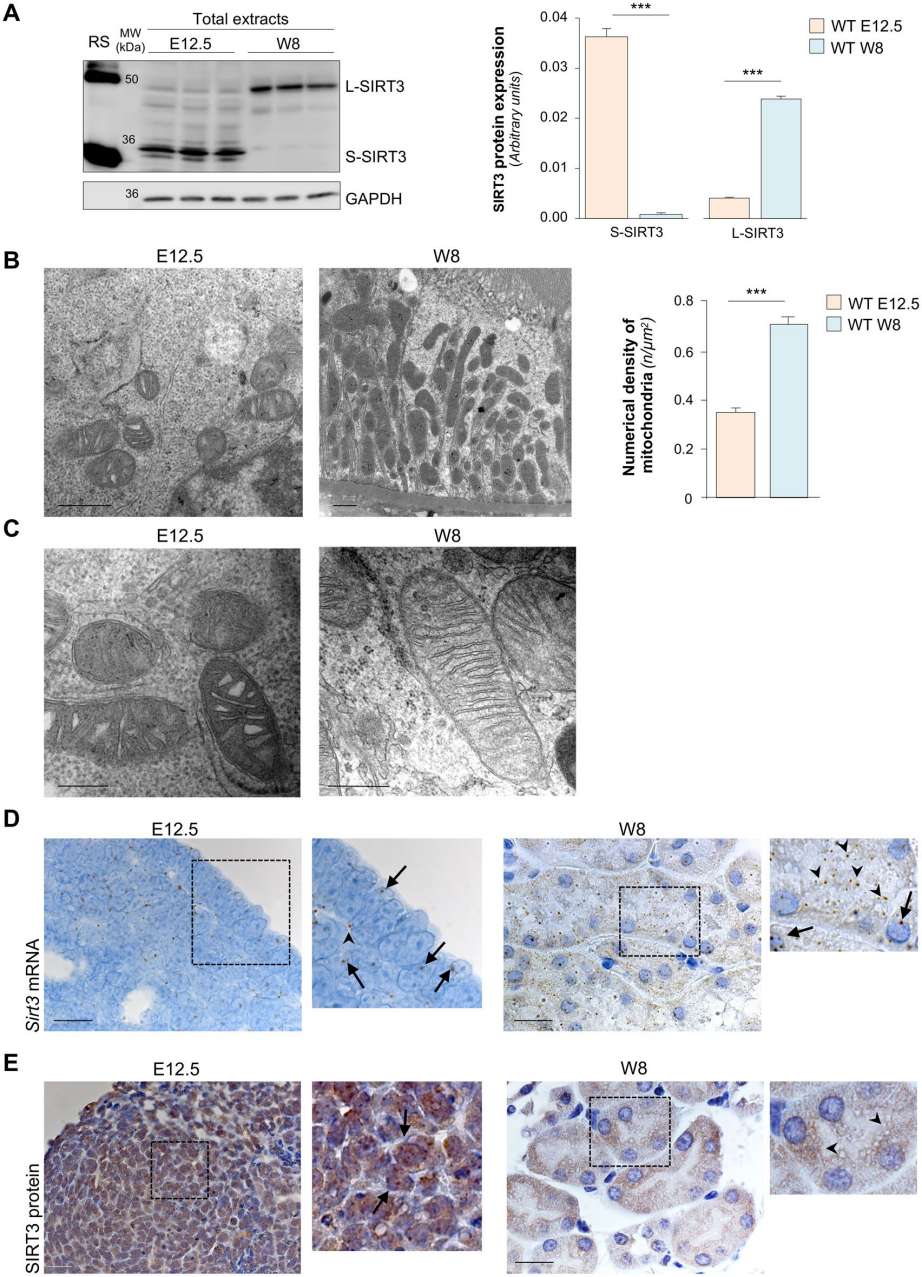
SIRT3 expression and localisation during kidney development. (**A**) Representative Western blots and densitometric analysis of short (S-SIRT3, 28 kilo Dalton, kDa) and long (L-SIRT3, 44 kDa) isoform expression in total extracts of WT kidneys on embryonic day (E) 12.5 and week-8 (W8) kidneys (*n* = 3 samples per group). In the first lane, recombinant SIRT3 (RS) was used as a positive control for the identification of different SIRT3 isoforms. Molecular weights (MW) are reported in the second lane and expressed in kDa. For E12.5 extracts, each sample is the pool of 15 metanephroi. For W8 extracts, each sample is a single mouse kidney. GAPDH was used as sample loading control. (**B**) Representative transmission electron micrographs of mitochondrial ultrastructure and quantification of mitochondria numerical density in resin-embedded kidney sections from WT mice at E12.5 and W8 (*n* = 2 mice per group, 30 TEM pictures from each sample). Scale bars, 500 nm left panel, 1 µm right panel. (**C**) Representative electron micrographs of mitochondria at high magnification showing the presence of non-fused, round-shaped organelles with loose, non-aligned cristae randomly distributed in the mitochondrial matrix, suggestive of an immature morphology on E12.5 (left panel). Mitochondria in W8 kidney exhibited the characteristic mitochondrial morphology as documented by elongated organelles with highly packed and tight cristae within the matrix, with proximal tubular cells showing the highest mitochondrial content (right panel) (*n* = 2 mice per group). Scale bar, 250 nm. (**D**) Representative images of in situ hybridisation for *Sirt3* mRNA in kidney from WT mice on E12.5 and at W8. Insets show the localisation of *Sirt3* mRNA in nuclei (arrows) or cytoplasm (arrowheads) of renal progenitor cells on E12.5 and adult renal cells at W8 (*n* = 3 mice per group). Scale bar, 20 μm. (**E**) Representative immunohistochemistry images of SIRT3 protein expression on E12.5 and W8 kidneys. Insets show the localisation of SIRT3 protein in nuclei of renal progenitor cells on E12.5 (arrows), while SIRT3 protein expression is mainly localised in the cytoplasm (arrowheads) of adult renal cells at W8 (*n* = 3 mice per group). Scale bar, 20 μm. Data represent mean ± s.e.m. and were analysed with Student’s *t*-test. ***P < 0.001.

To elucidate the role of SIRT3 in kidney development, we first assessed SIRT3 protein expression in murine kidneys isolated on embryonic day (E) 12.5 compared to postnatal week (W) 8. The two different isoforms of SIRT3 (28 kDa short and 44 kDa long isoform) were studied. Western blot analysis revealed that, compared to W8, renal tissues on E12 exhibited significantly higher levels of the short SIRT3 isoform, whereas no expression of the long SIRT3 isoform was observed (Fig. 1A). At W8, the long isoform was the most prevalent (Fig. 1A). To gain deeper insight into the predominant expression of SIRT3 during embryonic development, we analysed mitochondria that have been reported as the main site of SIRT3 localization in adult cells. Transmission electron microscopy (TEM) and morphometric analysis revealed significantly lower mitochondrial density at E12.5, assessed as number of mitochondria per µm^2^, compared to W8 kidneys (Fig. 1B). The relatively low content of mitochondria on E12.5 was associated with the presence of non-fused, round organelles with loose, non-aligned cristae randomly distributed in the mitochondrial matrix (Fig. 1C), suggesting immature morphology. At variance, mitochondria in the adult kidney exhibited the characteristic mitochondrial morphology as documented by elongated organelles with highly packed and tight cristae within the matrix in proximal tubular cells (Fig. 1C).

To explain the high levels of SIRT3 expression at E12.5 in the face of few immature mitochondria, we analysed the SIRT3 mRNA and protein expression pattern. In the embryonic renal tissue, cells exhibited an extremely large nucleus that occupied most of the cell body (Fig. 1D,E). Through in situ hybridisation experiments, we found that on E12.5 *Sirt3* mRNA had a localization that was predominantly nuclear (Fig. 1D, arrows) and a less frequent extra-nuclear arrangement (Fig. 1D, arrowheads). In the adult renal cells, at W8 *Sirt3* mRNA was visible in some nuclei (Fig. 1D, arrow), although it was mainly expressed in the extra-nuclear compartment (Fig. 1D, arrowheads). Immunohistochemistry analysis confirmed the widespread expression of SIRT3 in metanephroi on E12.5, particularly but not exclusively in the nucleus (Fig. 1E), while SIRT3 was mostly confined to the extra-nuclear compartment and the perinuclear region in adult cells at W8 (Fig. 1E).

**Figure 2:**
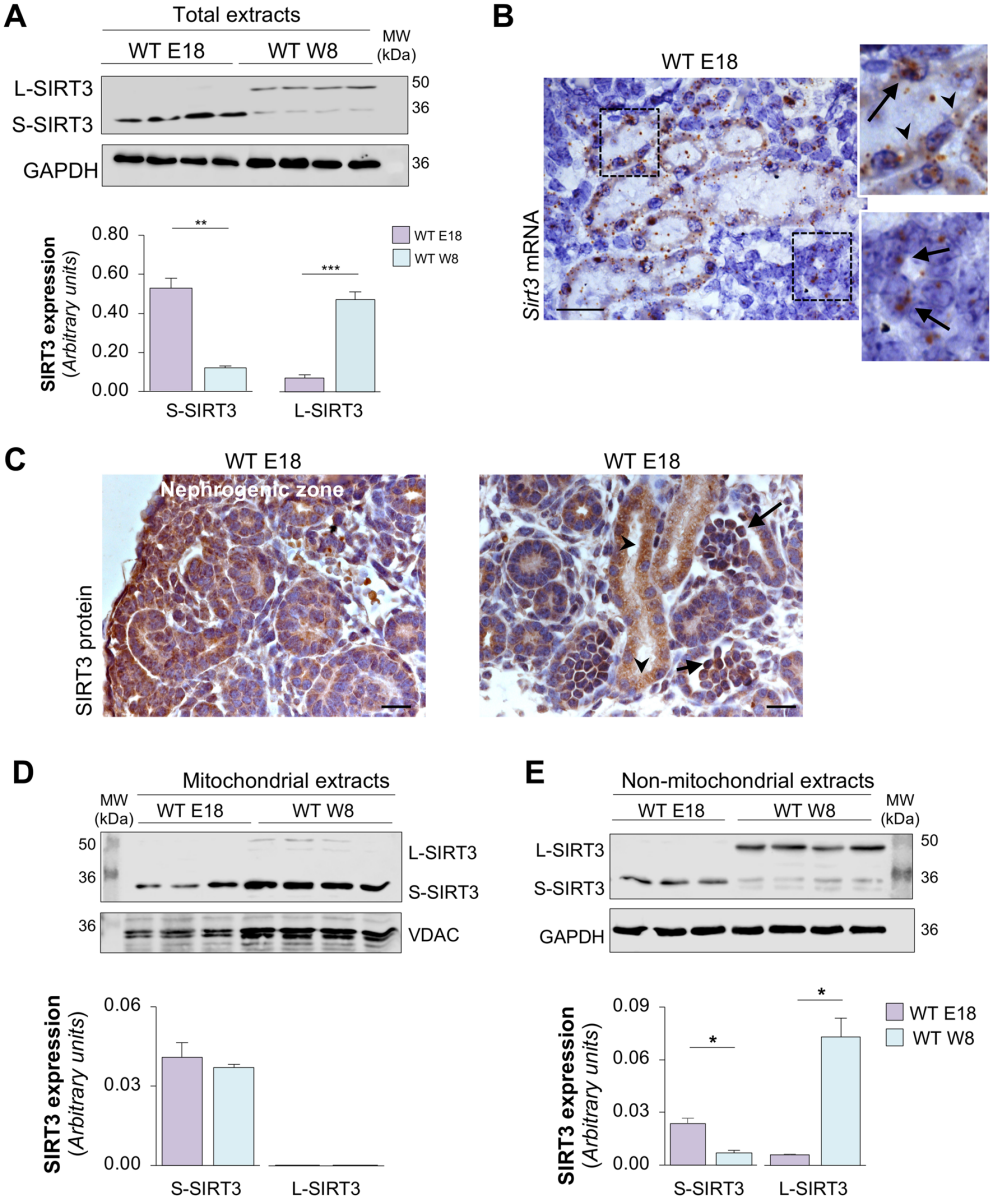
SIRT3 expression pattern changes during different kidney maturation stages. (**A**) Representative Western blots and densitometric analysis of S-SIRT3 and L-SIRT3 isoform expression in total extracts of kidneys on E18 and at W8 (*n* = 4 samples per group). For E18 extracts, each sample is the pool of 5 kidneys. For W8 extracts, each sample is a single mouse kidney. GAPDH was used as sample loading control. (**B**) Representative images of in situ hybridisation for *Sirt3* mRNA in kidney from WT mice on E18. Insets show the localisation of *Sirt3* mRNA in the cytoplasm (arrowheads) and nuclei (arrows) of more differentiated renal structures while *Sirt3* mRNA expression is mainly localised in the nuclei of still immature structures (arrows, *n* = 3 mice per group). Scale bar, 20 μm. (**C**) Representative immunohistochemistry images of SIRT3 protein expression on E18 showing high nuclear SIRT3 expression in the nephrogenic zone, localised in the outer kidney cortex (left panel) and in immature structures (arrows, right panel), as opposed to the cytoplasmic localisation of SIRT3 in developed tubular structures, possibly reflecting a mitochondrial localisation (arrowheads, right panel*; n* = 3 mice per group). Scale bar, 20 μm. (**D**,**E**) Representative Western blots and densitometric analysis of the expression of SIRT3 isoforms in (**D**) isolated mitochondrial and (**E**) non-mitochondrial extracts harvested from WT kidney on E18 and at W8 (*n* = 3 samples per group). For E18 extracts, each sample is the pool of 5 kidneys. For W8 extracts, each sample is a single kidney. Voltage-dependent anion-selective channel 1 (VDAC) and GAPDH were used as sample loading controls for mitochondrial and non-mitochondrial extracts, respectively. Molecular weights (MW) are reported in each representative Western Blot and expressed in kDa. Data represent mean ± s.e.m. and were analysed with Student’s *t*-test. *P < 0.05, *P < 0.01, and ***P < 0.001.

To further substantiate the different localisation of SIRT3 during the developmental stages of the kidney, we analysed SIRT3 expression patterns at an intermediate time during embryonic development (E18), when both immature and already mature renal structures are present. At this developmental stage, we found that the expression of the short SIRT3 isoform was higher in total extracts from E18 kidneys than in the postnatal kidney at W8 (Fig. 2A). On E18, renal extracts did not express the long SIRT3 isoform which, on the contrary, was expressed at higher levels in W8 (Fig. 2A). Through in situ hybridisation, we found that on E18 cells in more immature structures exhibited a pronounced nuclear mark of *Sirt3* mRNA (Fig. 2B, lower inset; arrows). Otherwise, in cells within more developed tubular structures, *Sirt3* mRNA was mainly localized in the extra-nuclear compartment (Fig. 2B, upper inset; arrowhead), although a nuclear localization was also identifiable in few cells (Fig. 2B, upper inset; arrows). Immunohistochemistry analysis revealed that on E18 SIRT3 protein was highly expressed in the outer cortex, the nephrogenic zone (Fig. 2C, left panel), possibly reflecting the pivotal role of SIRT3 during renal cell maturation. In the internal areas of the kidney, nuclear localization of SIRT3 protein was particularly noticeable in more immature structures (Fig. 2C, right panel; arrow), while extra-nuclear localization was especially evident in the more developed tubular compartment (Fig. 2C, right panel; arrowheads). To support these observations, we performed western blot analysis in separate cellular fractions, namely the mitochondrial and non-mitochondrial extracts. In mitochondria, SIRT3 was only expressed as a short isoform (S-SIRT3) and its expression in the organelles was similar in the developing kidney on E18 and in the adult kidney at W8 (Fig. 2D). Conversely, the non-mitochondrial expression of SIRT3 exhibited a different pattern, with the short SIRT3 isoform being the most widely expressed form on E18, while SIRT3 was mainly expressed as a long isoform (L-SIRT3) at W8 (Fig. 2E). Given the low mitochondrial content on E12.5 and the consequent technical difficulties with isolating them, it was not possible to perform an analysis of SIRT3 protein expression on fractionated E12.5 tissues. Otherwise, on E12.5, SIRT3 was only expressed as a short isoform as shown in Fig. 1A.

To investigate the actual contribution of SIRT3 to regulating mitochondrial development during renal embryogenesis, we studied mice deficient for *Sirt3*. In this setting, TEM analysis revealed that the immature morphology of mitochondria on E12.5 was not affected by the lack of SIRT3 (Supplementary Fig. S1A). In contrast, ultrastructural analysis on E18 showed a higher percentage of mitochondria with disorganized cristae (52%) in *Sirt3*^*−/−*^ than in WT renal cells (1.58%) (Supplementary Fig. S1B, higher magnification). Following complete mitochondrial maturation in W8, *Sirt3*^*−/−*^ mice exhibited round and smaller mitochondria compared to their WT littermates, which is reflected in the reduced mean mitochondrial volume (Supplementary Fig. S1C), in spite of unaffected mitochondrial density (WT, 0.659 ± 0.03 n/μm^3^, n = 3; *Sirt3*^*−/−*^*,* 0.673 ± 0.07 n/μm^3^, n = 4; mean ± s.e.m.).

Collectively, these findings suggest that SIRT3 expression patterns change during different maturation stages of the kidney. In the developing kidney, SIRT3 is expressed exclusively as a short isoform, particularly in the nucleus. In adult renal tissues, SIRT3 short isoform is restricted to mitochondria, while the long isoform is present in the non-mitochondrial extracts, including the nucleus.

### SIRT3 has de-2-hydroxyisobutyrylase activity, affecting post-translational modifications of histones during renal development

**Figure 3:**
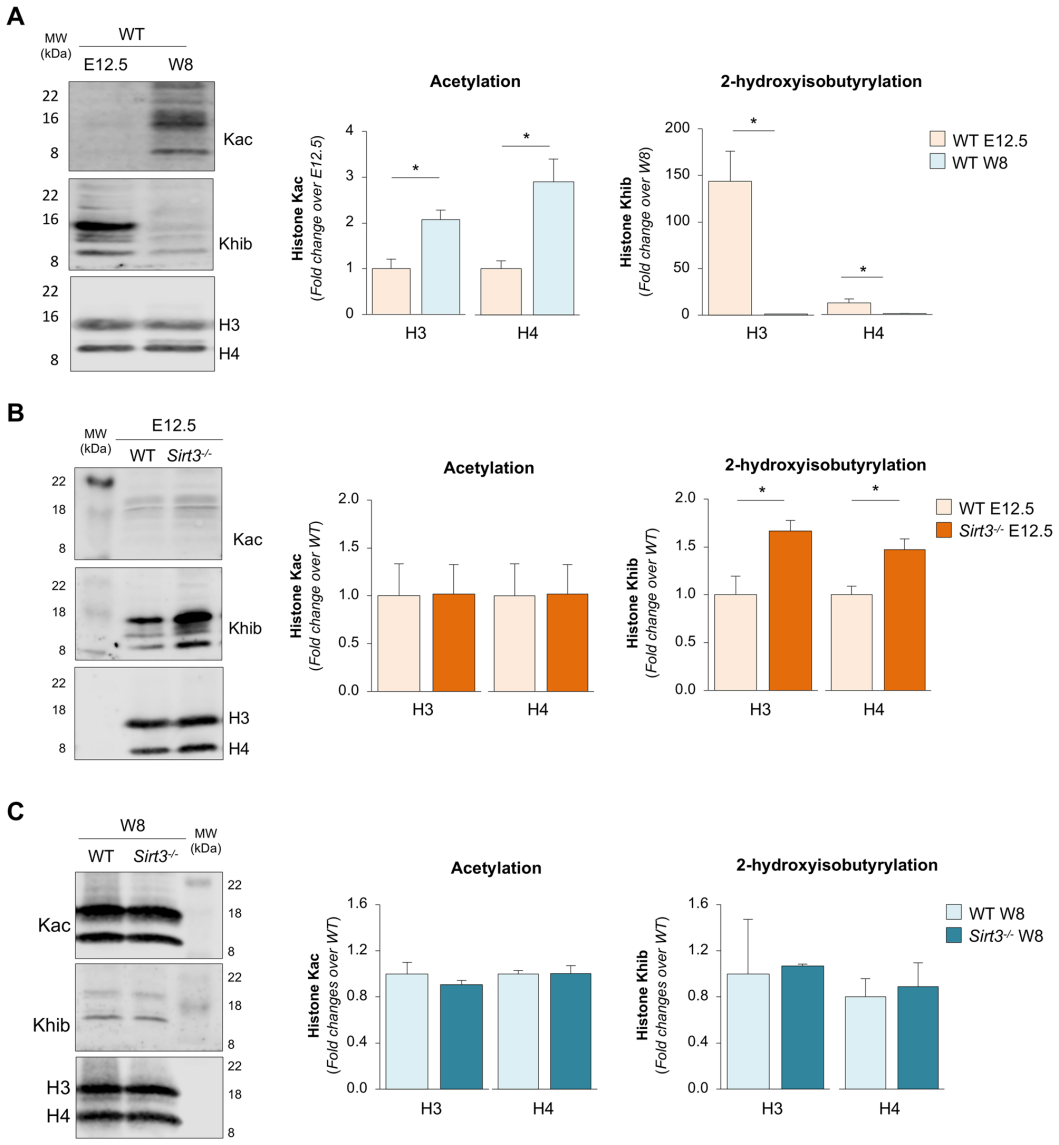
SIRT3 regulates histone 2-hydroxyisobutyrylation in the embryonic kidney. (**A**) Representative Western blots and densitometric analysis of histone H3 (H3) and H4 lysine acetylation (Kac) or lysine 2-hydroxyisobutyrylation (Khib) in total extracts harvested from WT kidneys on E12.5 and at W8 (*n* = 3 samples per group). For E12.5 extracts, each sample is the pool of 15 metanephroi. For W8 extracts, each sample is a single kidney. (**B**) Representative Western blots and densitometric analysis of H3 and H4 Kac or Khib in total extracts harvested from WT and *Sirt3*^*−/−*^ kidneys on E12.5 (*n* = 3 samples per group, each sample is the pool of 15 metanephroi). (**C**) Representative Western blots and densitometric analysis of H3 and H4 Kac or Khib in total extracts harvested from WT and *Sirt3*^*−/−*^ kidneys at W8 (*n* = 3 mice per group). Molecular weights (MW) are reported in each representative Western Blot and expressed in kDa. Data represent mean ± s.e.m. and were analysed with Student’s t-test. *P < 0.05.

Previous studies have shown, mainly in vitro, that SIRT3 may reside in the nucleus, acting as a histone deacetylase during stress-related stimuli^20,21,35^. For this reason, we sought to evaluate whether nuclear SIRT3, present in the short isoform, could act as a histone deacetylase in vivo during renal development by analysing the co-localisation of a pan anti-acetyl lysine antibody and anti-histone antibodies, which reflect *bona fide* SIRT3 nuclear activity. Western blot analysis showed that acetylation on lysine residues (Kac) of histone H3 and H4 was barely detectable in total extracts of renal cells on E12.5 (Fig. 3A). The contribution of SIRT3 to renal Kac of histone was studied by using mice deficient for *Sirt3*. Kac of histone H3 and H4 was no higher in metanephroi isolated from *Sirt3*^*−/−*^ mice than in metanephroi harvested from WT littermates (Fig. 3B). Similarly, at W8 we found that the high levels of H3 and H4 Kac (Fig. 3A) were not affected by the *Sirt3* deficiency (Fig. 3C). These data indicate that SIRT3 does not affect post-translational modification of histones through acetylation, either on E12.5, where it is predominantly in the short nuclear isoform, or at W8 when it is present in the nuclei mainly as a long isoform.

A very recent study has indicated that SIRT3 displays a peculiar lysine-2-de-hydroxyisobutyrylase activity on H3 and H4 in human kidney cells in vitro^36^. Lysine-2-hydroxyisobutyrylation (Khib) is a unique epigenetic mark on specific lysine residues that is associated with active gene transcription in meiotic and post-meiotic cells^37^. Thus, we assessed whether SIRT3 exhibits a histone de-2-hydroxyisobutyrylase activity during kidney development by investigating the histone Khib. Western blot analysis showed that Khib levels of histone H3 and H4 were markedly high in total extracts of renal cells on E12.5 compared to W8 (Fig. 3A). By analysing E12.5 metanephroi from *Sirt3*^*−/−*^ mice, we found that H3 and H4 Khib was regulated by SIRT3, as indicated by their significant increase compared to WT counterparts (Fig. 3B). Conversely, at W8 the low levels of histone Khib were not affected by *Sirt3* deficiency (Fig. 3C).

Overall, these data suggest that short nuclear SIRT3 plays a role in controlling histone Khib on E12.5, while the long nuclear SIRT3 in adult kidneys at W8 lacks de-2-hydroxyisobutyrylase activity.

### SIRT3 de-2-hydroxyisobutyrylase activity regulates glycolysis during renal development

**Figure 4:**
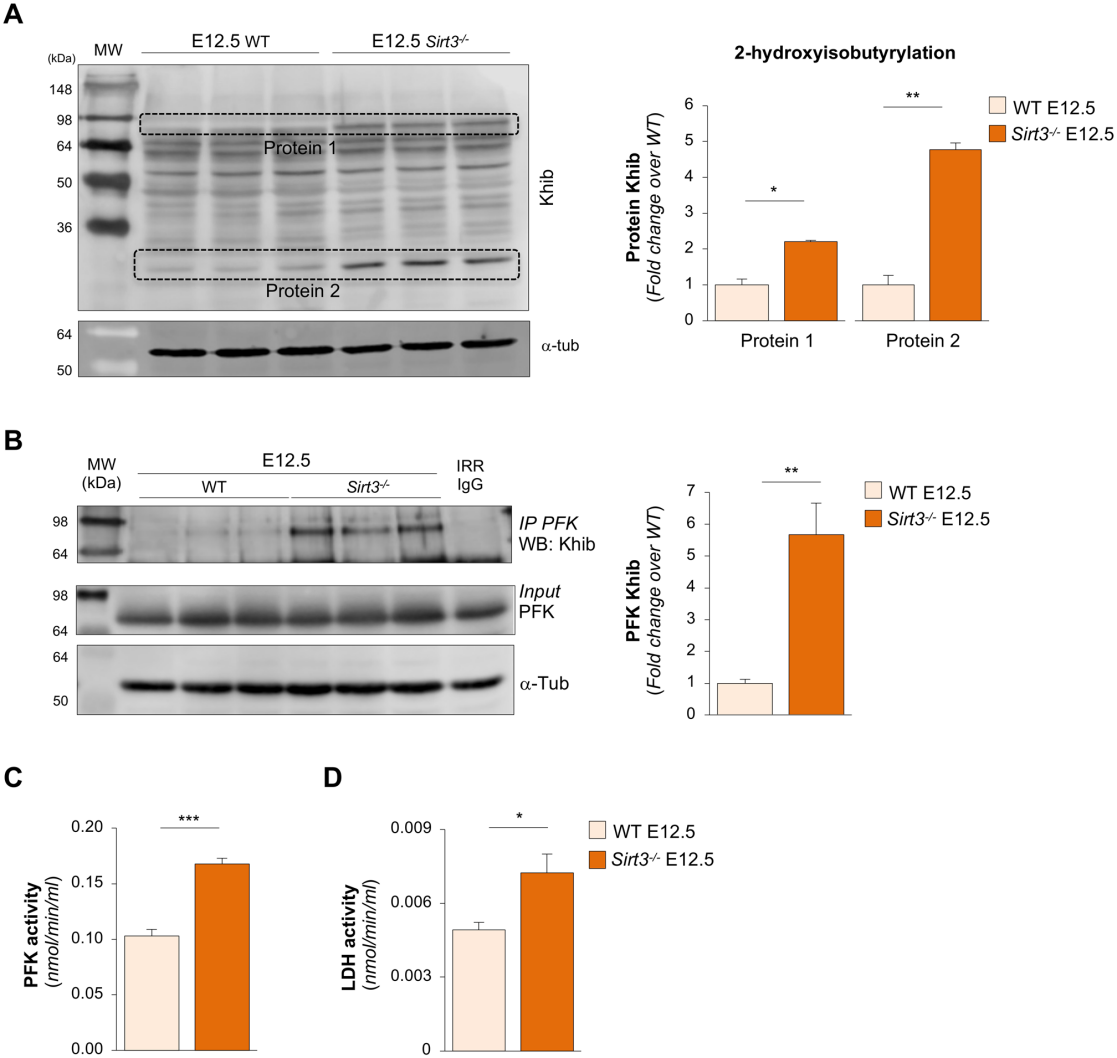
SIRT3 regulates glycolysis in the embryonic kidney via 2-hydroxyisobutyrylation. (**A**) Representative Western blots of lysine 2-hydroxyisobutyrylation (Khib) on non-histone proteins in total extracts harvested from WT and *Sirt3*^*−/−*^ kidneys on E12.5 (*n* = 3 samples per group, each sample is the pool of 15 metanephroi). α-tubulin (α-tub) was used as sample loading control. Quantification of the Khib levels of two specific proteins (dotted boxes) found to be hyper-Khib in *Sirt3*^*−/−*^ kidneys on E12.5. (**B**) Western Blot (WB) of Khib in total extracts from WT and *Sirt3*^*−/−*^ kidneys on E12.5 immunoprecipitated phosphofructokinase (PFK). Normal rabbit IgG was used as an irrelevant isotype control (IRR IgG). Input identifies the basal expression of PFK and normalized for α-tub as sample loading control by WB in total extracts from WT and *Sirt3*^*−/−*^ kidneys on E12.5 (*n* = 3 samples per group, each sample is the pool of 15 metanephroi). (**C**) PFK activity assed by colorimetric assay kit in total extracts from WT and *Sirt3*^*−/−*^ kidneys on E12.5 (*n* = 4 sample per group, each sample is the pool of 10 metanephroi). (**D**) Lactate dehydrogenase (LDH) activity assessed in total extracts from WT and *Sirt3*^*−/−*^ kidneys on E12.5 (*n* = 6 sample per group, each sample is the pool of 15 metanephroi). Molecular weights (MW) are reported in each representative Western Blot and expressed in kDa. Data represent mean ± s.e.m. and were analysed with Student’s t-test. *P < 0.05, **P < 0.01, and ***P < 0.001.

In addition to histones, several non-histone proteins have been found to be regulated by Khib^38,39^. Therefore, we sought to evaluate the effects of SIRT3 on the level of Khib on non-histone proteins in embryonic kidneys by analysing the expression of the pan lysine-2-hydroxyisobutyrylation in total protein extracts on E12.5. Using western blot analysis, we found that two specific proteins—with an approximate molecular weight of 80 kDa (protein 1) and 30 kDa (protein 2)—had significantly higher levels of Khib in metanephroi lacking *Sirt3* compared to WT counterparts (Fig. 4A). Given that the most widely characterised Khib mark on non-histone protein has been found on glycolytic enzymes^38,39^, we focused on this metabolic pathway as the possible target for the de-2-hydroxyisobutyrylase activity of SIRT3 on E12.5. In particular, we studied phosphofructokinase (PFK), which has a molecular weight of 80 kDa, possibly corresponding to the hyper-2-hydroxyisobutyrylated protein 1 found in *Sirt3*^*−/−*^ mice. To confirm that PFK is the target for SIRT3 de-2-hydroxyisobutyrylase activity, we performed immunoprecipitation experiments to evaluate the levels of Khib on PFK. As shown in Fig. 4B, we found that PFK exhibited a significant increase in Khib levels in embryonic kidneys of *Sirt3*^*−/−*^ mice on E12.5 compared to WT metanephroi. To evaluate the functional relevance of Khib on PFK, we assessed the activity of PFK in renal tissue. We found that hyper-Khib of PFK in *Sirt3*^*−/−*^ kidney was associated with increased PFK activity compared to their WT littermates on E12.5 (Fig. 4C).

Previous studies showed that increased Khib levels on glycolytic enzymes are associated with higher glycolytic flux^38,39^. To evaluate whether the hyper-Khib levels and the increased activity of PFK had a functional effect on glycolysis in our setting, we investigated the activity of lactate dehydrogenase (LDH), which is the final step of the glycolytic pathway. To this end, we performed a colorimetric activity assay that detects the reduction of NAD to NADH catalysed by LDH and found that *Sirt3* deficiency in E12.5 metanephroi led to a significant increase in the activity of LDH and glycolytic flux (Fig. 4D). Unlike in the embryonic kidney, the altered Khib pattern in total protein extracts at W8 was not affected by *Sirt3* deficiency, as confirmed by similar levels of Khib on protein 1 and 2 compared to SIRT3 competent mice (Supplementary Fig. S2A).

Taken together, all the above suggests that the SIRT3 short isoform on E12.5 plays a prominent role in the regulation of Khib of glycolytic enzymes during early development.

### Sirt3 deficiency does not affect the redox state of the embryonic kidney

During development, the kidney is exposed to hypoxia and to a balance of pro- and anti-oxidant pathways that are fine-tuned to govern gene expression and nephrogenesis^40^. Since SIRT3 has been shown to regulate a cluster of proteins involved in the regulation of cellular redox state^41,42^, we studied the expression of the mitochondrial superoxide dismutase (SOD2), a ROS scavenging enzyme directly targeted by SIRT3^43,44^. As shown in Supplementary Fig. S2B, SOD2 was not detectable in the embryonic kidney, while its expression significantly increased in the adult tissue. At W8, we found that *Sirt3*^*−/−*^ deficiency resulted in the increase in Kac of SOD2 compared to WT littermates (Supplementary Fig. S2B). More recently, optic atrophy 1 (OPA1) has been shown to be a critical regulator of redox state, as loss of functional OPA1 induces a pro‐oxidative state in the cell^45^. Having identified in our previous studies that OPA1 is a direct target for the deacetylase activity of SIRT3^28,46^, we sought to investigate OPA1 Kac in our experimental setting. We found that OPA1 expression is detectable in embryonic kidney (Supplementary Fig. S2C), although Kac levels were not affected by *Sirt3* deficiency (Supplementary Fig. S2C). Conversely, a significant increase in OPA1 Kac was found in *Sirt3*^*−/−*^ mice compared to WT littermates at W8 (Supplementary Fig. S2C).

Having identified a different SIRT3-dependent regulation pattern of antioxidant enzymes between embryonic and adult kidneys, we evaluated nitrotyrosine, a marker of protein oxidation^28^, in our experimental setting. As shown in Supplementary Fig. S3, comparable high nitrotyrosine expression was detected in WT and *Sirt3*^*−/−*^ kidneys isolated on E18. Conversely, in the adult renal tissue we found that nitrotyrosine expression significantly increased in *Sirt3*^*−/−*^ mice compared to WT mice (Supplementary Fig. S3).

Collectively, these data suggest that, conversely to what observed in the mature kidneys, during development, SIRT3 does not exert significant deacetylase activity on selected mitochondrial proteins that can regulate oxidative stress in the organelle. Moreover, given the similarly high levels of nitrotyrosine in WT and *Sirt3*-deficient mice, oxidative stress is presumably not involved in impaired nephrogenesis of *Sirt3*-deficient mice.

### Sirt3 deficiency affects the self-renewal of renal progenitor cells

**Figure 5:**
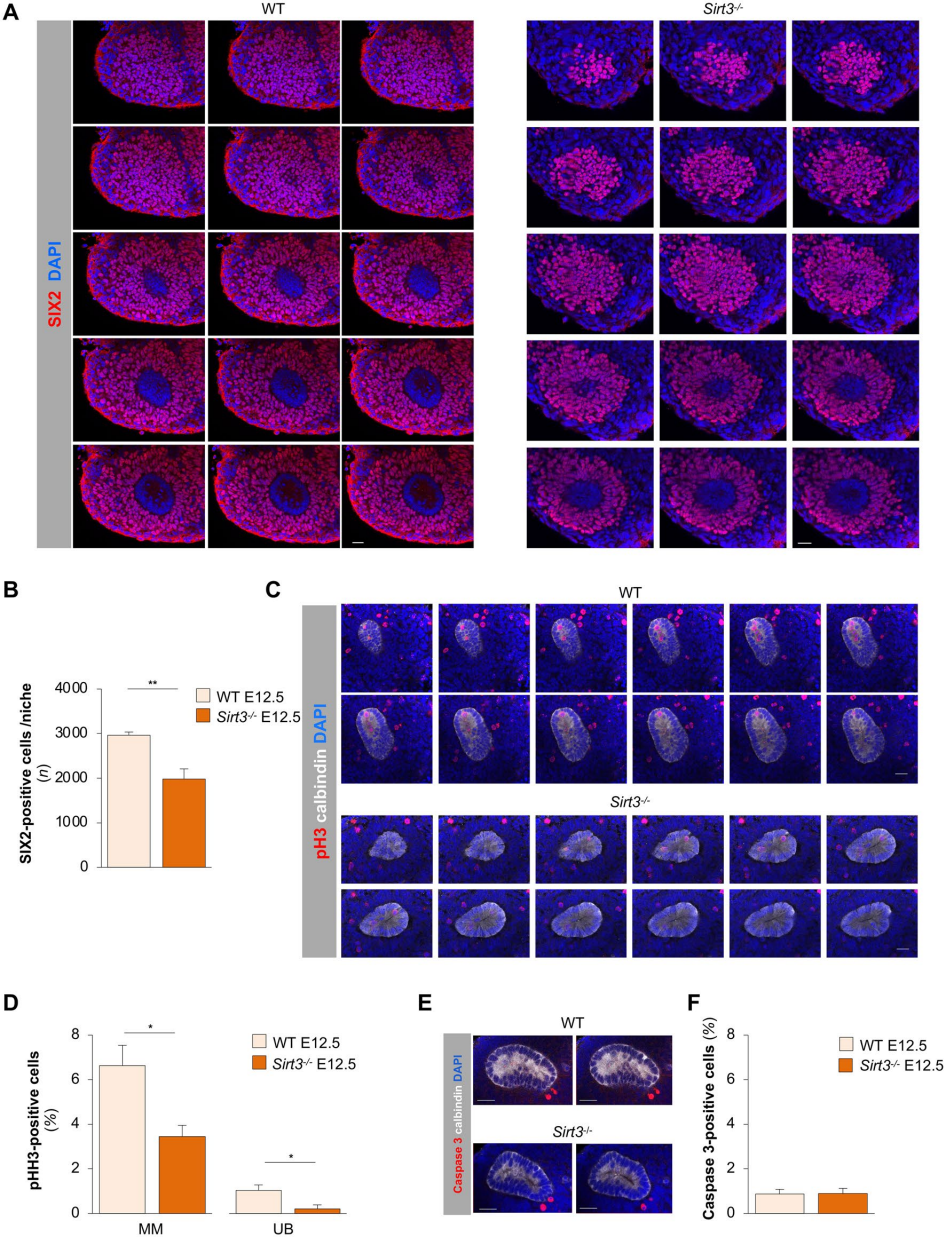
SIX2-positive progenitor cell number and proliferation decrease in *Sirt3*^*−/−*^ metanephroi. (**A**) Representative confocal z stack series of individual niche of E12.5 metanephroi stained for SIX2 (red) and DAPI (blue). Scale bars, 20 µm. (**B**) Quantification of the numbers of SIX2-positive cells in E12.5 metanephroi in WT and *Sirt3*^*−/−*^ mice. SIX2-positive cells were counted in z stack optical sections acquired through each single niche (*n* = 5 mice per group). (**C**) Representative confocal z stack series of individual niche of E12.5 metanephroi stained for phospho-Histone H3 (pH3, red), calbindin (white) and DAPI (blue). Scale bars, 20 µm. (**D**) Quantification of cell proliferation in metanephric mesenchyme (MM) and UB of E12.5 metanephroi in WT and *Sirt3*^*−/−*^ mice. The percentage of phospho-histone H3 (pH3)-positive cells has been quantified in whole metanephros (*n* = 6 mice per group). (**E**) Representative images of two different z-stack sections of E12.5 metanephroi stained for Caspase 3 (red), calbindin (white) and DAPI (blue). Scale bars, 20 µm. (**F**) Quantification of apoptosis in E12.5 metanephroi from WT and *Sirt3*^*−/−*^ mice by counting Caspase 3-positive cells (*n* = 6 mice per group). Data represent mean ± s.e.m. and were analysed with Student’s *t*-test. *P < 0.05, and **P < 0.01.

Glycolysis is the major metabolic pathway used by renal progenitor cells and is a cell-intrinsic determinant of their fate^47–49^. Therefore, we sought to explore the impact of SIRT3-dependent regulation of histone post-translational modification and glycolysis on nephrogenesis by studying renal progenitor cells in mice deficient for *Sirt3*. To this end, we elected to study SIX2 which, throughout mammalian kidney development, identifies a multipotent progenitor cell population in the metanephric mesenchyme controlling the differentiation towards different epithelia of the nephron^5,6,50,51^. We performed whole mount staining on E12.5 to investigate whether *Sirt3* deficiency might be associated with alterations in the nephron progenitor pool of the MM. The number of SIX2-positive cells was quantified per niche, defined as the cap mesenchyme surrounding each adjacent tip^32^, by z-stack optical section. We found that in *Sirt3*^−/−^ mice the number of SIX2-positive renal progenitor cells per niche was 33% lower than in WT mice (Fig. 5A,B).

To understand whether changes in cell proliferation and/or apoptosis were responsible for the lower number of progenitor cells in *Sirt3*^−/−^ kidneys, we studied phospho-Histone H3 (pH3) and cleaved caspase 3 expression in whole E12.5 metanephroi. The analysis of z stack optical sections showed that the percentage of pH3-positive cells was significantly higher in the MM of WT mice compared to *Sirt3*^−/−^ mice. In addition, proliferating cells in the UB compartment, identified by calbindin staining, was reduced in mice lacking *Sirt3* (Fig. 5C,D). The quantification of cell apoptosis in WT mice on E12.5 showed there was a low percentage of apoptotic cells, which was comparable to that observed in *Sirt3*^−/−^ mice (Fig. 5E,F). The impact of *Sirt3* deficiency on the nephrogenic area was still considerable on E18. Indeed, in *Sirt3*^*−/−*^ mice, the nephrogenic area, identified by the presence of SIX2-positive progenitor cells, exhibited a significant reduction in thickness as compared to WT mice (Supplementary Fig. S4A,B).

To further assess what effect *Sirt3* deficiency has on renal cell precursor behaviour and activity, we studied the duration of nephrogenesis, which normally ceases by postnatal day 3 (P3) in mice^52,53^, by analysing the presence of SIX2-positive cells in the kidneys on P3 and P4. In *Sirt3*^−/−^ mice, SIX2-positive cells persisted over P3, whereas in WT kidneys they had already disappeared (Supplementary Fig. S4C). On P4, no SIX2-positive cells were found in either WT or *Sirt3*^*−/−*^ kidneys (Supplementary Fig. S4C).

Altogether, these results indicate that *Sirt3* deficiency reduces the number of SIX2 progenitor cells and impairs cell proliferation both in the MM and UB. Moreover, the lack of SIRT3 is associated with the prolonged presence of SIX2-positive cells in newborn kidneys at the expense of timely renal maturation, suggesting an important contribution of SIRT3 in the regulation of embryonic kidney development.

### Sirt3 deficiency affects nephrogenesis and causes a nephron number deficit that persists throughout adulthood

**Figure 6:**
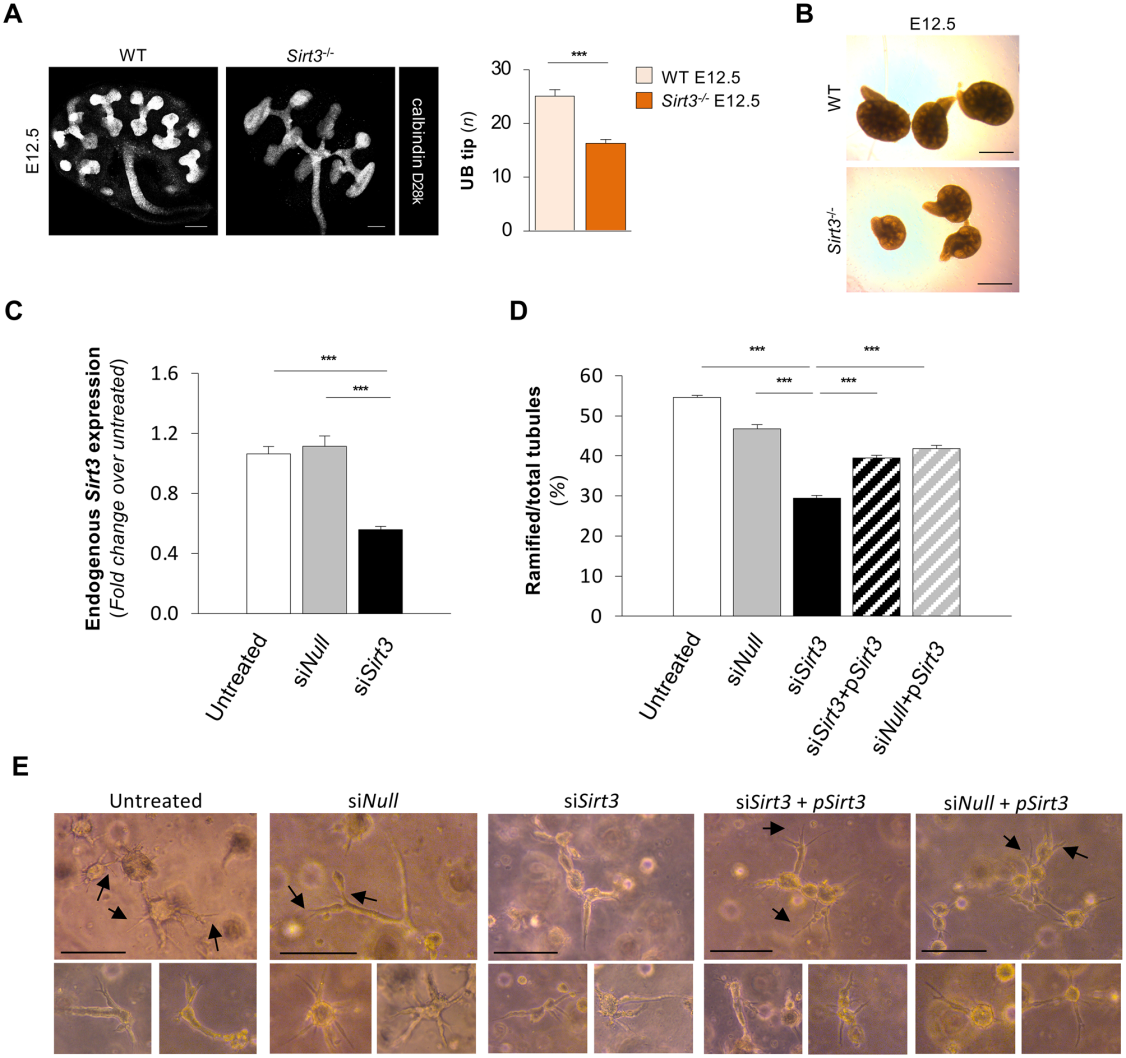
*Sirt3* deficiency alters ureteric bud branching in vivo and impairs in vitro tubulogenesis. (**A**) Representative immunofluorescence images of calbindin D28k-positive ureteric bud (UB) trees of E12.5 kidneys (representative of *n* = 3 mice per group) and quantification of UB tips per kidney in WT and *Sirt3*^*−/−*^ mice on E12.5 (*n* = 17, WT and *n* = 12, *Sirt3*^*−/−*^ mice). Scale bars, 100 μm. (**B**) Representative pictures of kidneys from WT or *Sirt3*^*−/−*^ mice on E12.5 under contrast phase microscope. Scale bars, 500 μm. (**C**) Expression of endogenous *Sirt3* mRNA by real-time PCR in untreated mIMCD3 cells, mIMCD3 transfected with a control nontarget small interfering RNA (si*Null*), or with a silencer select predesigned small interfering mouse *Sirt3* (si*Sirt3*) (n = 3 independent experiments). (**D**) Quantification of mIMCD3 cell-derived ramified tubules per total tubules in untreated, si*Null* or si*Sirt3* cells transfected or not with a vector overexpressing the short *Sirt3* isoform (p*Sirt3*). n = 3 samples per group. For each experimental group, the number of total fields analysed were: n = 55 for untreated, n = 50 for si*Null*, n = 58 for si*Sirt3*, n = 53 for si*Sirt3* + p*Sirt3*, and n = 49 for si*Null* + p*Sirt3*. (**E**) Representative images of tubules formed in a 3D collagen culture system. At 3 days, untreated and si*Null* cells give rise to complex and ramified tubules, some of which exhibit terminal bifid and trifid branching (arrows). si*Sirt3* cells form mainly straight tubules and small round clusters of cells. The ability of tubules to branch was re-stablished in si*Sirt3* cells by plasmid-mediated *Sirt3* overexpression. Scale bars, 100 μm. Data represent mean ± s.e.m. and were analysed with Student’s *t*-test or ANOVA corrected with Bonferroni coefficient, as appropriate. ***P < 0.001.

To understand whether the altered renal progenitor cell behaviour induced by a *Sirt3* deficiency could result in impaired nephrogenesis and nephron number, we studied the morphogenesis of the ureteric bud (UB) on E12.5, using calbindin D28k staining. The results revealed a smaller UB tree, reduced branching, and a significant reduction in the number of UB tips (Fig. 6A) in *Sirt3*^−/−^ mice compared to WT mice. As a result, the kidneys of E12.5 *Sirt3*^−/−^ mice were markedly smaller than those of their WT littermates (Fig. 6B). Similarly, in an ex vivo assay, *Sirt3*^−/−^ kidneys (E11.5) maintained in culture for up to 10 days exhibited developmental retardation compared to WT mice, which was most evident between 1 and 3 days of culture (Supplementary Fig. S5). To further confirm that SIRT3 contributes to UB branching morphogenesis, we used mIMCD3 cells silenced for *Sirt3* by small interfering (siRNA) approaches that exhibited almost the 50% reduction in *Sirt3* expression (Fig. 6C) and cultured them in a 3D collagen-based system that allows for in vitro tubulogenesis^33,54^. Remarkably, *Sirt3-*silenced cells gave rise to a significantly lower number of ramified tubules, indicated by arrows, compared to si*Null* and extensively formed cell clusters (Fig. 6D,E).

To provide evidence that delivery of the SIRT3 short isoform can restore the ability of mIMCD3 to develop ramified tubules when the endogenous *Sirt3* is silenced, we overexpressed short *Sirt3* isoform by plasmid transfection (p*Sirt3*). Through this maneuver, exogenous *Sirt3* protein was abundantly expressed, as revealed by low Ct values compared to those of untreated cells (Ct values, untreated: undetectable *vs* si*Null* + p*Sirt3*: 20.45 ± 0.097) in spite of unchanged levels of endogenous *Gapdh* (Ct values, untreated: 18.32 ± 0.015 *vs* si*Null* + p*Sirt3*: 18.62 ± 0.023). Endogenous *Sirt3* silencing did not affect the expression of exogenous *Sirt3* (Ct values, si*Null* + p*Sirt3*: 20.45 ± 0.097 vs si*Sirt3* + p*Sirt3*: 18.79 ± 0.010). In this setting, plasmid-mediated *Sirt3* overexpression was able to significantly re-establish the ability of the cells to form branching tubules (Fig. 6D,E; arrows).

Then, we sought to investigate whether impaired tubulogenesis in mIMCD3 cells silenced for *Sirt3* was dependent on alterations in the Khib levels of glycolytic enzymes, as observed in the in vivo setting. To this end, we performed immunoprecipitation experiments that revealed a significant increase in the Khib levels of PFK in cells silenced for *Sirt3* compared to si*Null* cells (Supplementary Fig. S6A), which was accompanied by a significant increase in the activity of PFK (Supplementary Fig. S6B).

**Figure 7:**
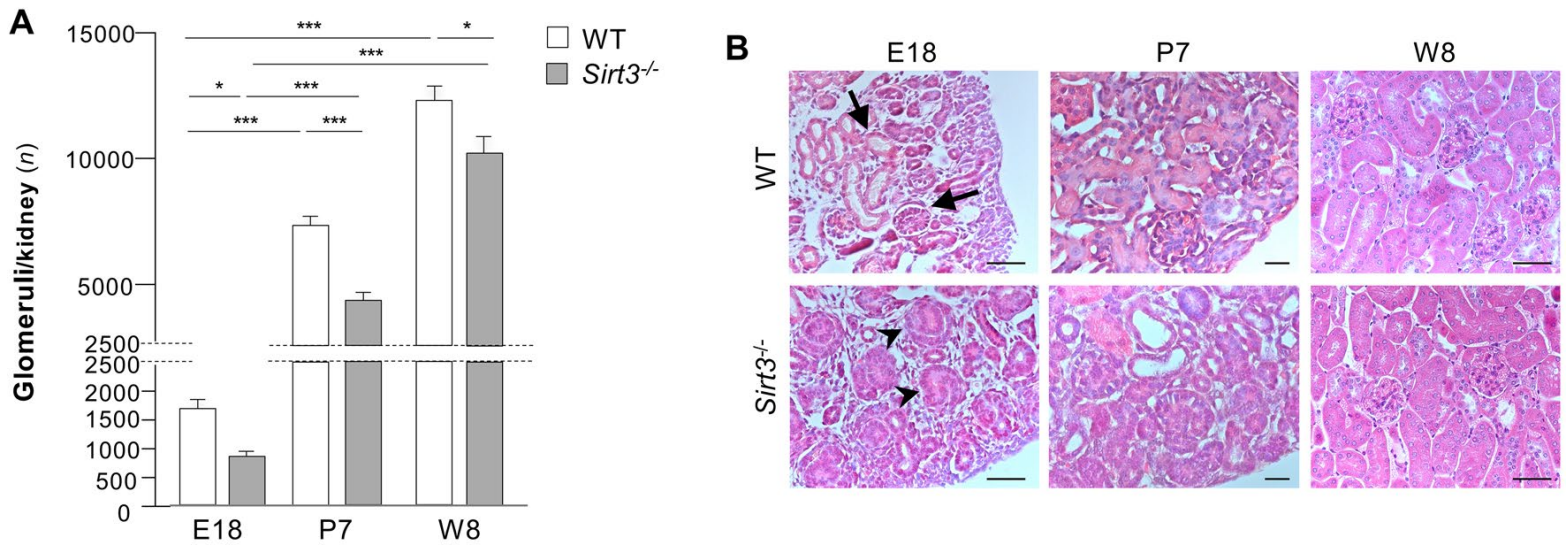
*Sirt3*^*−/−*^ mice exhibit a nephron number deficit. (**A**) Quantification of the number of glomeruli per kidney in WT and *Sirt3*^*−/−*^ mice on E18, postnatal day 7 (P7), and W8 (*n* = 6 mice, WT and *Sirt3*^*−/−*^ on E18; *n* = 8 mice*,* WT and *Sirt3*^*−/−*^ on P7; *n* = 6 mice, WT and *n* = 4 mice, *Sirt3*^*−/−*^ in W8). (**B**) Histologic analysis of kidneys from E18 and postnatal (P7, P12 and W8) WT or *Sirt3*^*−/−*^ mice (representative of *n* = 3 mice per group). In WT mice, developed glomerulus and tubule are indicated by arrows. In *Sirt3*^*−/−*^ mice, arrowheads indicate immature structures. Scale bars, 20 μm for E18 and W8; 50 μm for P7. Data represent mean ± s.e.m. and were analysed with ANOVA corrected with Bonferroni coefficient. *P < 0.05, and ***P < 0.001.

The evidence that SIRT3 regulates renal development prompted us to investigate whether its deletion could impact nephrogenesis. To this end, we quantified the number of glomeruli, which reflects the nephron number in total kidneys, through renal tissue dissociation. On E18, kidneys from *Sirt3*^−/−^ mice had a significantly lower number of glomeruli than those of WT mice, which persisted on postnatal day 7 (P7) and W8 (Fig. 7A). At birth, neither the body nor kidney weights of *Sirt3*^*−/−*^ mice were statistically different from those of WT mice (body weight: WT 1.38 ± 0.04 and *Sirt3*^−/−^ mice 1.41 ± 0.3 g; kidney weight: WT 0.008 ± 0.001 and *Sirt3*^−/−^ mice 0.008 ± 0.001 g; mean ± s.e.m.).

The histological analysis on E18.5 showed that WT mice exhibited more developed kidneys with well-differentiated glomeruli and tubules (Fig. 7B; arrow), while immature structures were confined to the narrow nephrogenic area (Fig. 7B). In *Sirt3*^−/−^ mice, immature renal structures were more frequent (Fig. 7B; arrowheads) and distributed over a large area throughout the innermost part of the embryonic kidney, which exhibited very few well-developed glomeruli and tubules (Fig. 7B). However, no macroscopic differences could be observed on P7 and W8 between *Sirt3*^−/−^ kidneys and their WT (Fig. 7B).

These results indicate that SIRT3 is indispensable for appropriate nephrogenesis because it affects the behaviour of both MM and UB cells and is the key factor in determining nephron endowment and supporting the proper maturation of the renal compartments.

## Discussion

In this study, we discovered in the embryonic kidney that: (1) SIRT3 is highly expressed only as short isoform both in mitochondrial and the non-mitochondrial compartment, including the nucleus; (2) SIRT3 acts like a nuclear de-2-hydroxyisobutyrylase on lysine residues of histone proteins; (3) SIRT3 de-2-hydroxyisobutyrylase activity also affects non-histone proteins, particularly those of the glycolytic pathway, increasing their activity; (4) SIRT3-dependent regulation of these epigenetic programmes and metabolic pathways determines the course of nephrogenesis and final nephron number by affecting the renal cell precursor pool and behaviour.

To the best of our knowledge, our study is the first to report in vivo the presence of SIRT3 as a short isoform in the nucleus, and this expression pattern was found only during embryonic kidney development. This finding suggests that SIRT3 could potentially carry out post-translational modification activity on histones during the developmental process. Interestingly, we found that the nuclear SIRT3 short isoform did not possess a deacetylase activity on histones. Instead, we uncover that the SIRT3 short isoform regulated the lysine 2-hydroxyisobutyrilation (Khib) on histones in the nuclei of metanephroi. Remarkably, de-2-hydroxyisobutyrylase activity is unique to SIRT3 as it is not carried out by the classical nuclear sirtuins (SIRT 1, 2, 6 and 7), as recently reported in vitro^36^.

Khib is a novel evolutionarily conserved post-translational modification, which is likely derived from 2-hydroxyisobutyryl-coenzyme A (hib-CoA)^37^ and connects energy metabolism to epigenetic regulation^55,56^. The accumulation of Khib on the epsilon amino side-chain of lysine introduces a steric bulk and neutralises the positive charge of lysine^57^, resulting in active gene expression^38,39^. In prokaryotes, de-2-hydroxyisobutyrylation has been shown to be regulated by the NAD^+^‐dependent deacetylase CobB, which regulates glycolysis and cell growth^58^. In mammals, the enzymes acting as the “eraser” for Khib remain elusive^59^, while the lysine acetyltransferases p300 (KAT3B) has recently been identified as a “writer” for Khib-stimulating glycolysis in mammalian cells^38^. Our findings reveal the role that SIRT3 plays as an “eraser” for 2-hydroxyisobutyrylation in vivo during mammalian embryonic development. Indeed, using *Sirt3*^*−/−*^ mice, we found a significant increase in histone Khib, indicating that the SIRT3 short isoform plays a role in regulating epigenetics possibly affecting gene transcription during kidney development, a feature that has never been reported before. That SIRT3 displays a direct 2-de-hydroxyisobutyrylase activity has been convincingly documented by biochemical studies in human kidney cells in vitro^36^. However, it cannot be ruled out that lack of SIRT3 may induce alterations in metabolic pathway, which may in turn result in the alteration of the Khib profile observed in *Sirt3*^*−/−*^ embryonic kidneys.

Conversely, in the nuclei of adult renal cells SIRT3 is expressed as a long isoform. This finding is in line with previous reports showing that SIRT3 is localised in the nucleus as a long isoform and affects histone deacetylase activity^35,60,61^, especially in response to stress-mediated stimuli^20–22^. At variance with these studies, our results ruled out the possibility of any nuclear deacetylase activity of SIRT3 long isoform on histones in the adult kidney. Likewise, the nuclear SIRT3 long isoform does not exhibit de-2-hydroxyisobutyrylase activity. In mature renal cells, we found that the SIRT3 short isoform is exclusively localised in mitochondria acting as a deacetylase, as previously reported^26,62–65^. Future studies should be devoted to identifying the regulation of SIRT3 short isoform during kidney development, as well as the specific genes targeted by Khib during nephrogenesis.

In mammalian cells, Khib has also been shown to directly influence the properties of non-histone proteins, particularly glycolytic enzymes^38^. Here we found that, during early renal development, SIRT3 exerts de-2-hydroxyisobutyrylase activity on the glycolytic enzyme PFK, one of the rate limiting enzyme in the glycolytic pathway. In mammalian cells, an increase in Khib on glycolytic enzymes has been associated with a boost in their enzymatic activity^38^. Although we did not analyse lactate secretion to fully evaluate glycolytic flux, we found that hyper-Khib of PFK in *Sirt3*^*−/−*^ metanephroi at E12.5 was accompanied by a significant upsurge in glycolysis, as revealed by augmented LDH activity.

To date, several studies have documented the effect of metabolic pathway activity and nutrient availability on cell-fate-related outcomes, such as induced pluripotency^66,67^, maintenance of stemness^68–71^, and differentiation towards specific lineages^72–75^. In the attempt to evaluate the impact of the de-2-hydroxybutyrylase activity of SIRT3 on renal cell behaviour, we studied renal cell proliferation and nephron number in *Sirt3*^*−/−*^ mice. We highlighted that *Sirt3* deficiency is associated with an unexpected embryonic renal phenotype characterised by impaired nephrogenesis and decreased nephron number. Specifically, we found that *Sirt3*-deficient mice experienced a substantial impairment in UB branching and had a smaller nephron progenitor pool due to the decreased proliferation rate of cells of both the cap mesenchyme and UB tips, which impacts nephron number. Our data are in line with earlier evidence that suggests there is a functional link between early defects in branching morphogenesis, fewer nephrogenic progenitors and a nephron deficit^48,76^. Furthermore, we found that lack of SIRT3 prolonged nephrogenesis to the extent that SIX2-positive cells are present for a longer time in the post-natal kidneys. Glycolysis has been shown to be a pivotal, cell-intrinsic determinant for the fate of renal progenitor cells, with the inhibition of glycolysis stimulating their differentiation^48,77^. Consistently, our data suggest that the sustained glycolytic metabolism induced by the lack of SIRT3 results in altered differentiation of renal progenitor cells. Altogether, our results further our understanding in the molecular mechanisms underlying the complex interaction between Khib, epigenetics, and metabolic pathways in early embryonic development of the kidney.

In the perinatal period, newborns experience a drastic metabolic change from glycolysis to oxidative metabolism due to the switch from the anaerobic to aerobic respiration^78^. These changes in metabolic milieu could explain our findings on the ability of *Sirt3*-deficient mice to partially recover the nephron number which however, remains permanently reduced in adulthood by about 20% compared to WT mice. These findings could be particularly relevant in light of several observational studies in humans that have shown that abnormal programming of the kidney and reduced nephron number at birth predispose to renal diseases later in life^7–9^. Our earlier findings that *Sirt3-*deficient mice under resting conditions do not show altered renal function but, after acute kidney injury, exhibited more severe disease and died prematurely^28,79^, can be reconciled with our current study highlighting a defecting kidney development in mice lacking *Sirt3*. Our study could have potential translational relevance considering a recent evidence in humans showing that a deficiency of the essential SIRT3 co-factor NAD^+^, due to mutations in genes involved in its biosynthesis, results in congenital renal defects^80^.

## Conclusion

Collectively, all these data provide novel evidence that SIRT3 plays a regulatory role in kidney development by dictating renal progenitor proliferation and nephron endowment through post-translational modification of histone and non-histone proteins. These results may have important therapeutic implications, since they indicate that SIRT3 is a potential target for increasing nephron numbers and reducing the risk of renal disease later in life.

## Supplementary Information

Supplementary Figures.
