# Supplementary material for: Post-translational modifications by SIRT3 de-2-hydroxyisobutyrylase activity regulate glycolysis and enable nephrogenesis
Source: Sci Rep. 2021 Dec 8;11:23580. doi: 10.1038/s41598-021-03039-8 (PMC8655075; doi:10.1038/s41598-021-03039-8)
Supplement: Supplementary file 1 — Supplementary Figures. [file 41598_2021_3039_MOESM1_ESM.pdf]

Post-translational modifications by SIRT3 de-2-hydroxyisobutyrylase activity regulate glycolysis and enable nephrogenesis

Luca Perico,[1] Marina Morigi,[1] Anna Pezzotta,[1] Daniela Corna,[1] Valerio Brizi,[1] Sara Conti,[1] Cristina Zanchi,[1] Fabio Sangalli,[1] Piera Trionfini,[1] Sara Buttò,[1] Christodoulos Xinaris,[1] Susanna Tomasoni,[1] Carlamaria Zoja,[1] Giuseppe Remuzzi,[1] Ariela Benigni,[1] and Barbara Imberti[1*]

[1]Istituto di Ricerche Farmacologiche Mario Negri IRCCS, Bergamo, Italy

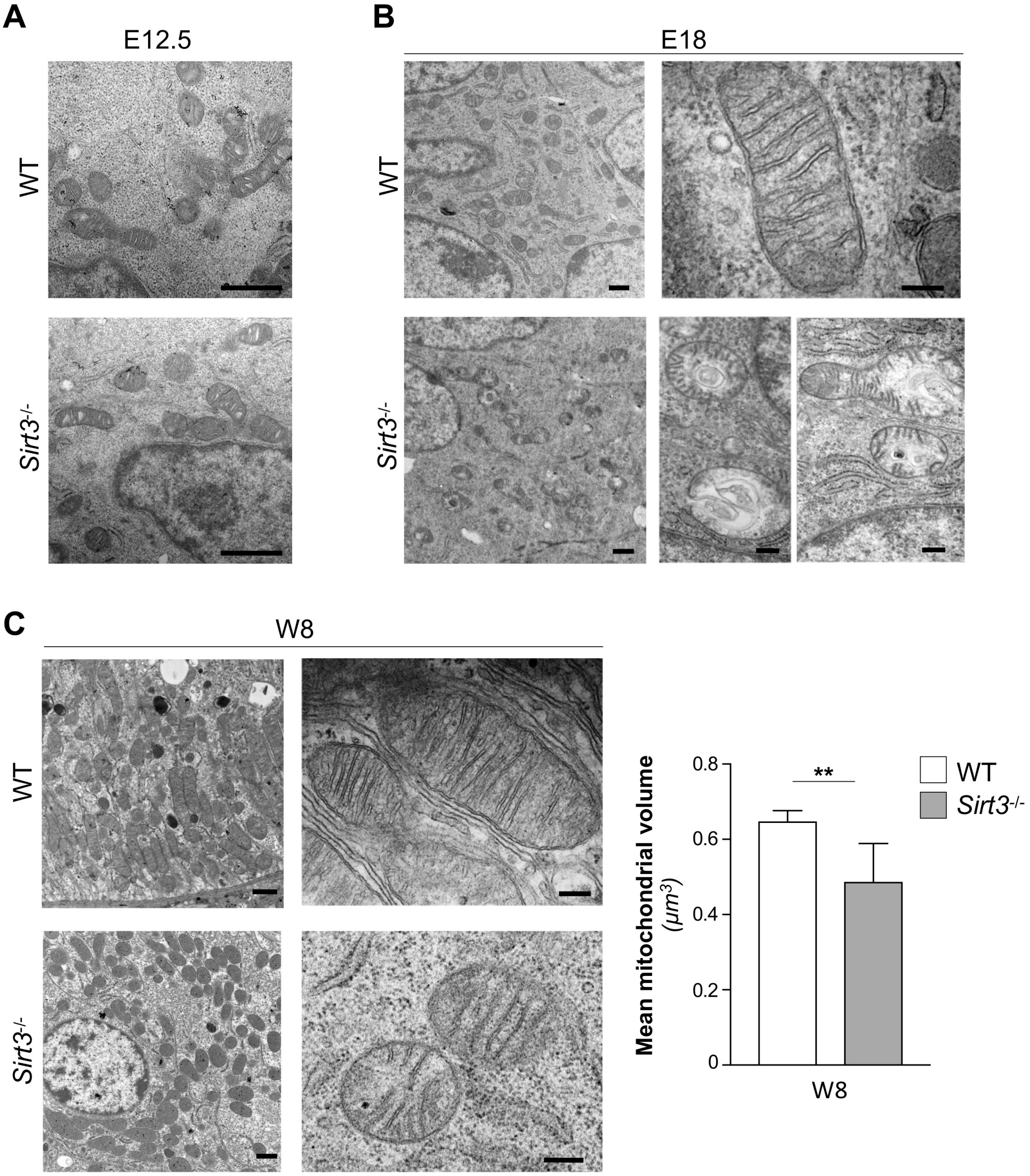

Figure S1. Ultrastructural analysis of mitochondria in renal cells from developing and adult kidneys. (A) Representative electron micrographs of mitochondria in embryonic renal cells of WT and *Sirt3*$^{-/-}$ mice on E12.5 (scale bars, 1 µm). **(B)** Representative electron micrographs of mitochondria at low (left panels; scale bars, 1 µm) and high magnification (right panels; scale bars, 200 nm) in WT and *Sirt3*$^{-/-}$ mice in embryonic renal cells on E18. **(C)** Representative electron micrographs of mitochondria in tubular cells of WT and *Sirt3*$^{-/-}$ mice at W8, at low (left panels; scale bars, 1 µm) and high magnification (right panels; scale bars, 200 nm). On the right, quantification of the mean mitochondrial volume in WT (n=3) and *Sirt3*$^{-/-}$ (n=4) mice at W8. Data represent mean ± s.e.m. and were analyzed by Student's t-test. ***P*<0.01.

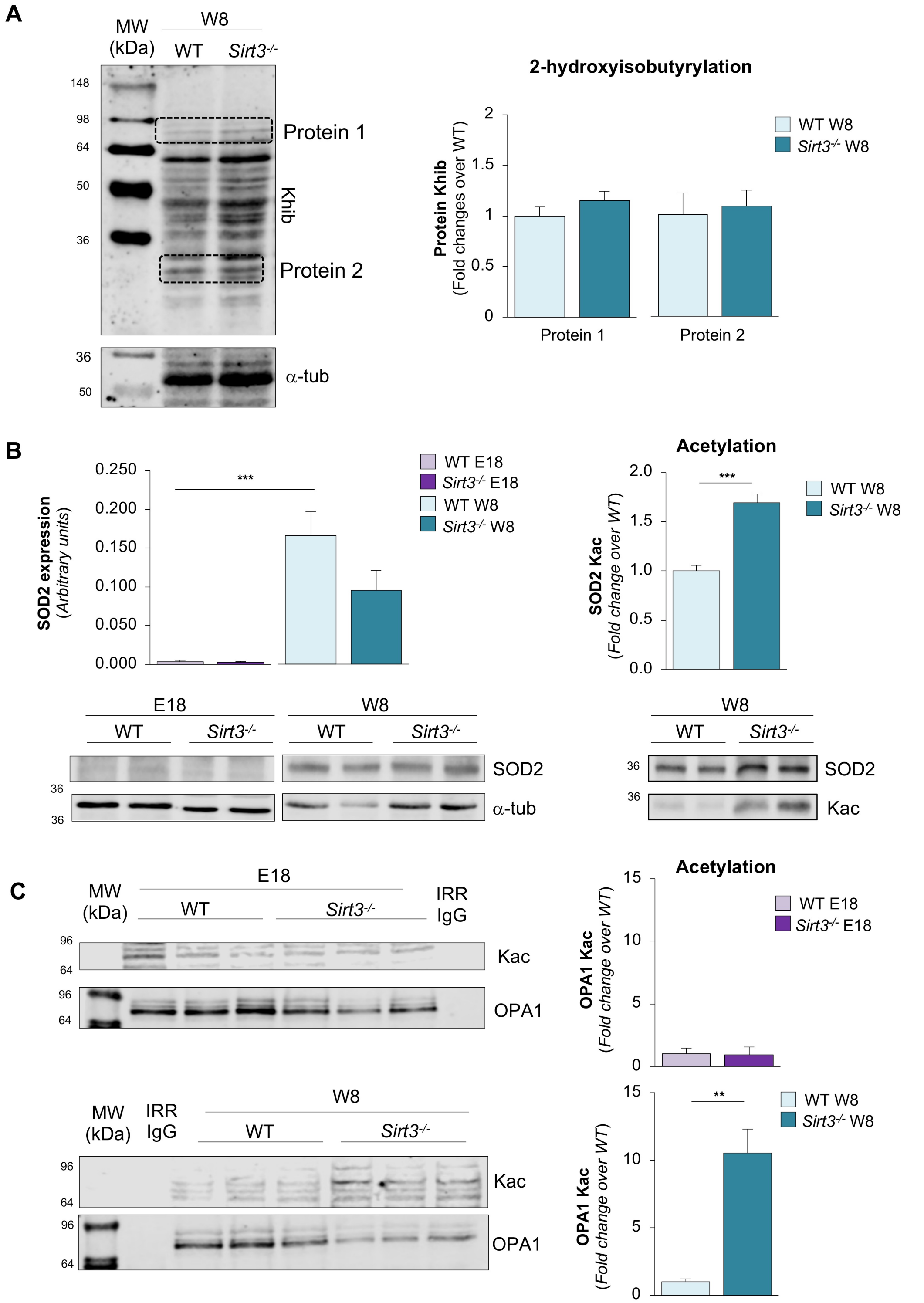

Figure S2. SIRT3 2-hydroxyisobutyrylation and acetylation activity in developing and adult kidneys. (**A**) Representative Western blots and densitometric analysis of lysine 2-hydroxyisobutyrylation (Khib) in total extracts harvested from WT and *Sirt3*$^{-/-}$ kidneys at W8 (*n*=3 mice per group). Dotted boxes represent the 2 hyper-Khib proteins identified to be upregulated in total renal extracts from *Sirt3*$^{-/-}$ metanephroi on embryonic day E12.5 shown in **Fig. 4A** and quantified on the left. α-tubulin (α-tub) was used as the sample loading control. (**B**) Densitometric analysis and representative Western blot of mitochondrial SOD2 expression and acetylation on lysine residues (Kac) in total extracts of WT or *Sirt3*$^{-/-}$ kidneys at E18 and W8 (*n*=4 mice per group). α-tub was used as the sample loading control. (**C**) Representative western blot and densitometric analysis of Kac of OPA1 by immunoprecipitation experiments in isolated mitochondria from renal tissues of WT and *Sirt3*$^{-/-}$ mice at E18 and W8 (*n*=3 mice per group). Normal rabbit IgG was used as an irrelevant isotype control (IRR IgG). Molecular weights (MW) are reported in each representative Western Blot and expressed in kDa. Data represent mean ± s.e.m. and were analyzed by Student's *t*-test or one-way ANOVA with Bonferroni's multiple comparisons test, as appropriate. ***P*<0.01, and ****P*<0.001.

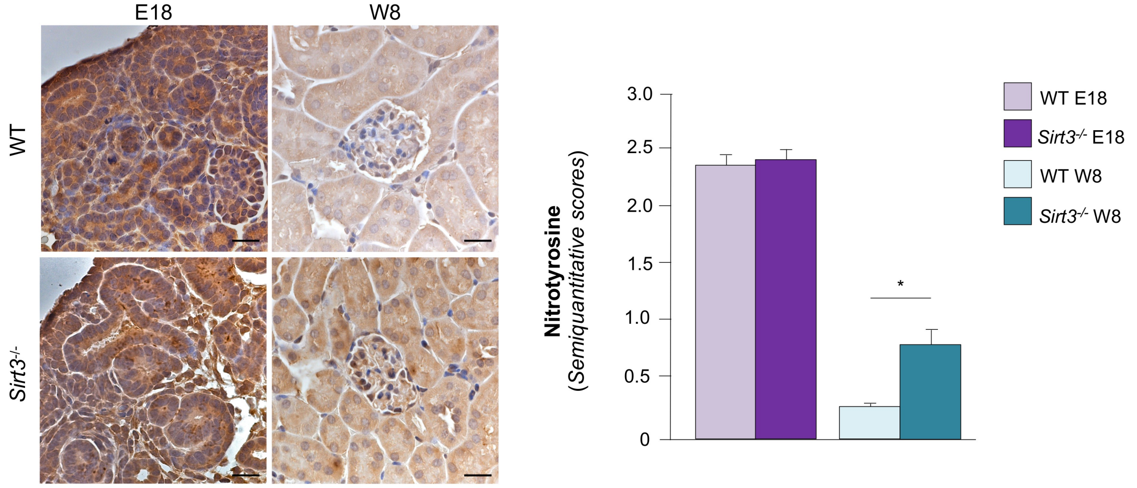

Figure S3. Immunohistochemical analysis of nitrotyrosine expression in renal tissues. Representative images and semiquantitative analysis of nitrotyrosine expression in tissues from E18 and W8 WT or *Sirt3*$^{-/-}$ kidneys (n=3 mice per group). Data represent mean ± s.e.m. and were analysed by one-way ANOVA with Bonferroni's multiple comparisons test. **P*<0.05. Scale bars, 20 μm.

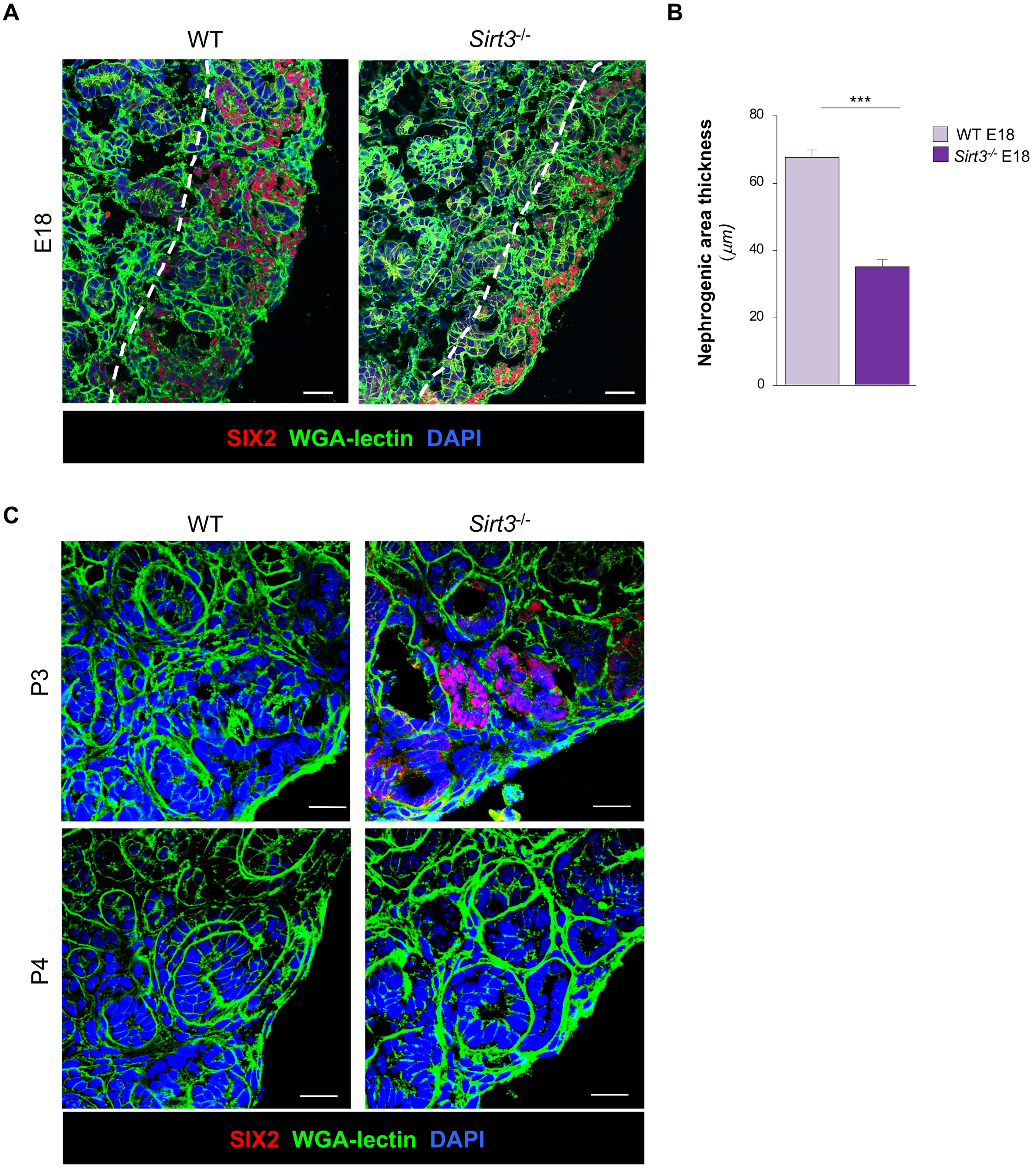

Figure S4. Expression of nephron progenitor marker SIX2 in kidneys at different developmental times. (A) Representative images of renal tissues, from E18 WT and *Sirt3*$^{-/-}$ mice, stained for SIX2 (red) WGA lectin (green) and DAPI (blue) (representative of n=3 mice per group). White dotted line indicates the limit where SIX2-positive cells are found within the tissue and indicates the boundary of the nephrogenic area. Scale bars, 25 μm. **(B)** Quantification of the thickness of the nephrogenic area in WT and *Sirt3*$^{-/-}$ mice on E18 tissues stained for SIX2 (red) WGA lectin (green) and DAPI (blue) (n=3 mice per group). **(C)** Representative images of SIX2-positive renal progenitors by immunofluorescence in WT and *Sirt3*$^{-/-}$ mice on postnatal day (P) 3 and P4 (representative of n=3 mice per group). Tissues are stained for SIX2 (red) WGA lectin (green) and DAPI (blue). Scale bars, 20 μm. Data represent mean ± s.e.m. and were analyzed by Student's t-test. ***P<0.001

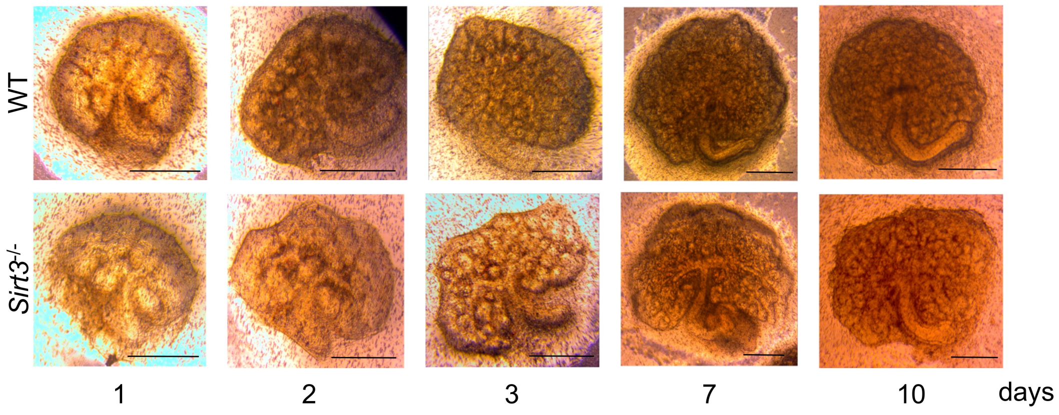

Figure S5. E*x vivo* culture of metanephroi from WT and *Sirt3*$^{-/-}$ mice. Phase contrast images of kidneys isolated from WT and *Sirt3*$^{-/-}$ mice on E11.5 grown in culture for ten days. *Sirt3*$^{-/-}$ kidneys exhibit a developmental retardation between 1-3 days of culture. Scale bars, 500 μm.

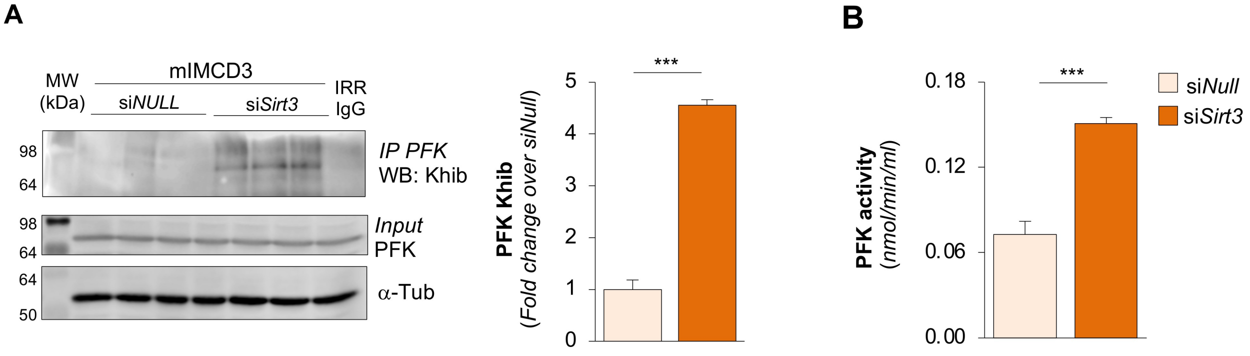

Figure S6. *Sirt3* regulates phosphofructokinase 2-hydroxyisobutyrylation and activity *in vitro*. (A) Representative Western Blot (WB) and quantification of lysine 2-hydroxyisobutirilation (Khib) of phosphofructokinase (PFK) in total extracts from mIMCD3 transfected with a control nontarget small interfering RNA (si*Null*) or with a silencer select predesigned small interfering mouse Sirt3 (si*Sirt3*) (n=3 independent experiments). Normal rabbit IgG was used as an irrelevant isotype control (IRR IgG). Molecular weight (MW) are reported on the left and expressed in kDa. WB in input identifies the basal expression of PFK in respect to sample loading control α-tubulin. **(B)** Analysis of PFK activity by colorimetric assay kit in total extracts from si*Null* and si*Sirt3* mIMCD3 (n=4 independent experiments). Data represent mean ± s.e.m and were analysed with Student's *t*-test. ****P*<0.001.

SIRT3 protein expression in Figure 1A

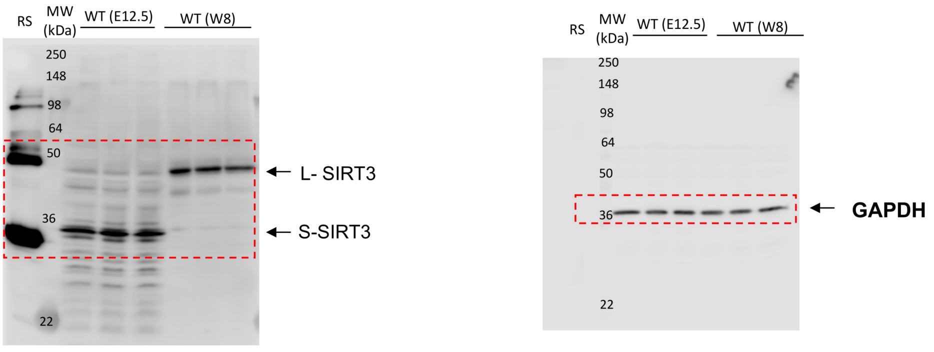

SIRT3 protein expression in Figure 2A

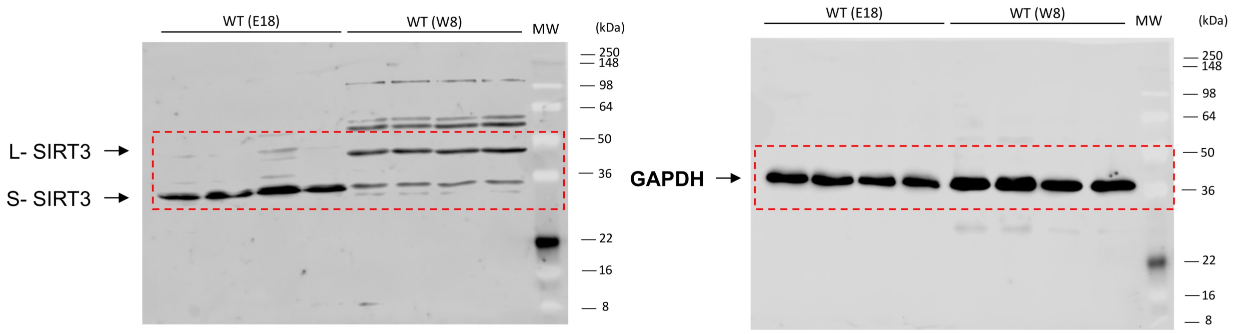

SIRT3 protein expression in Figure 2D

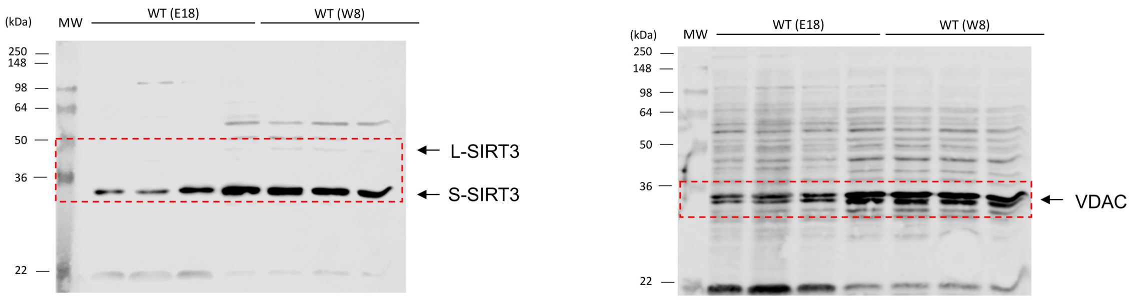

SIRT3 protein expression in Figure 2E

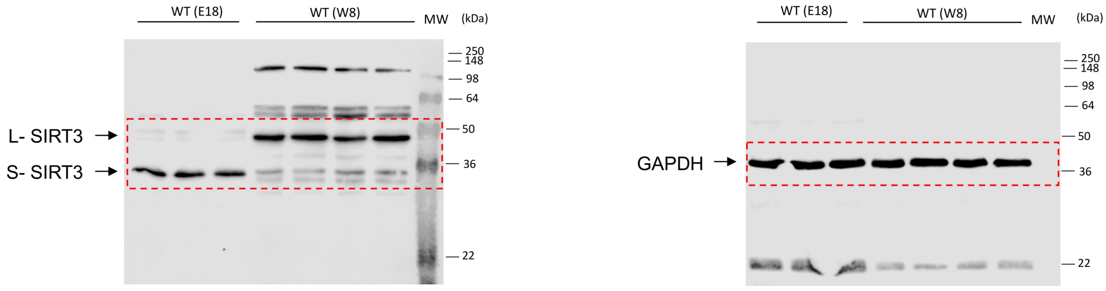

Figure S7. Uncropped gels of the protein expression of Sirtuin 3 (SIRT3) isoform (long isoform, L-SIRT3; short isoform, S-SIRT3) in Fig. 1A, 2A, 2D and 2E. Glyceraldehyde-3-phosphate dehydrogenase (GAPDH) and voltage-dependent anion-selective channel (VDAC) were used as sample loading control for total/cytoplasmic or mitochondrial extracts, respectively. Molecular weight (MW) are reported for each gel and expressed in kilo Dalton (kDa). WT, wild type; *Sirt3*$^{-/-}$, *Sirt3* knockout mice; E12.5, embryonic day 12.5; E18, embryonic day 18; W8, 8 weeks. Dotted red boxes indicates the portions of gel shown in the main figures of the manuscript.

Histone Kac in Figure 3A

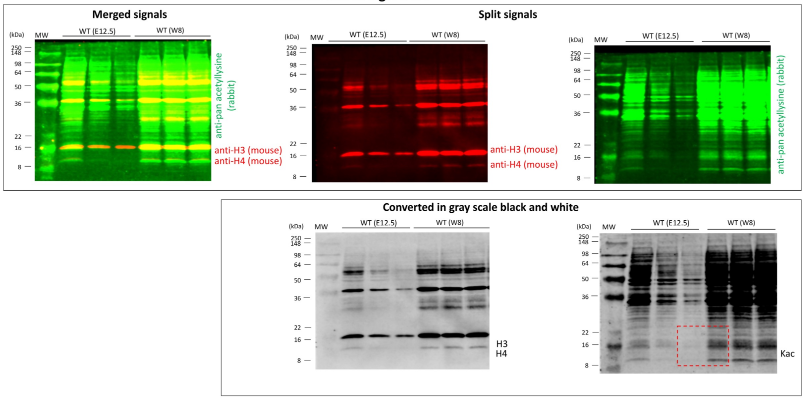

Histone Khib in Figure 3A

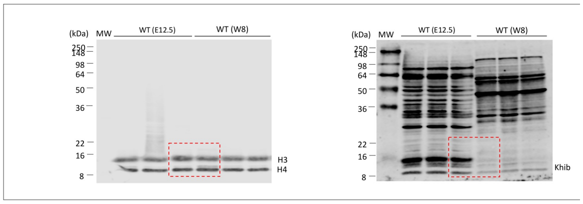

Histone Khib in Figure 3B

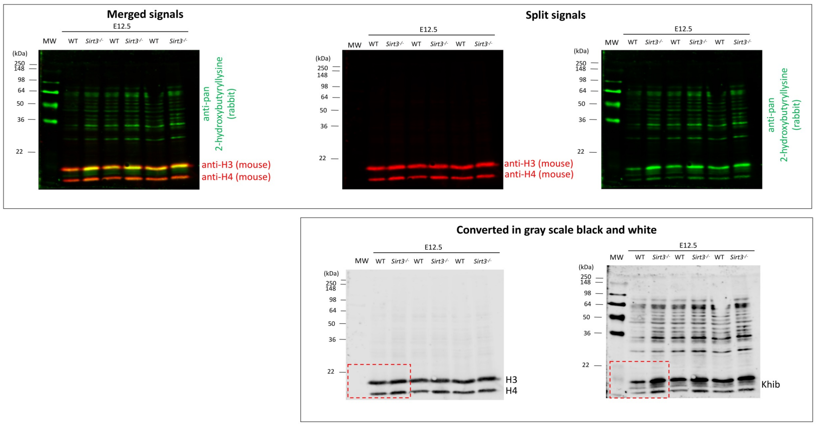

Histone Kac in Figure 3B

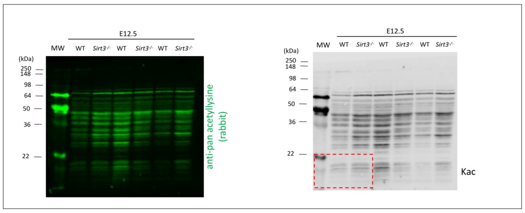

Histone Kac in Figure 3C

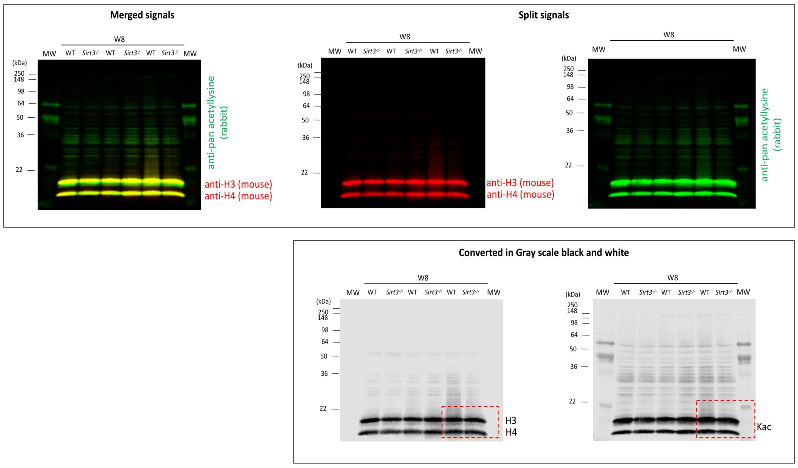

Histone Khib in Figure 3C

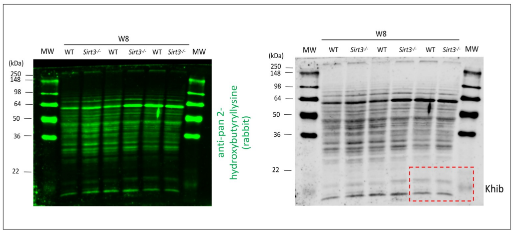

Figure S8. Uncropped gels of lysine acetylation (Kac) and lysine 2-hydroxyisobutyrylation (Khib) of histone (H)3 and H4 in Fig. 3A, 3B, and 3C. Molecular weight (MW) are reported for each gel and expressed in kilo Dalton (kDa). WT, wild type; *Sirt3*$^{-/-}$, *Sirt3* knockout mice; E12.5, embryonic day 12.5; W8, 8 weeks. Dotted red boxes indicates the portions of gel shown in the main figures of the manuscript.

A

Total protein Khib in Figure 4A

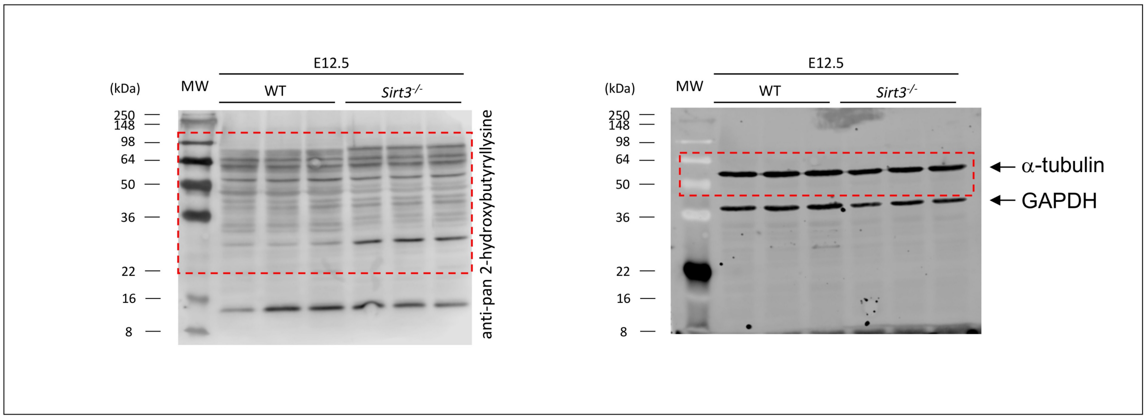

Total protein Khib in Supplementary Figure 2A

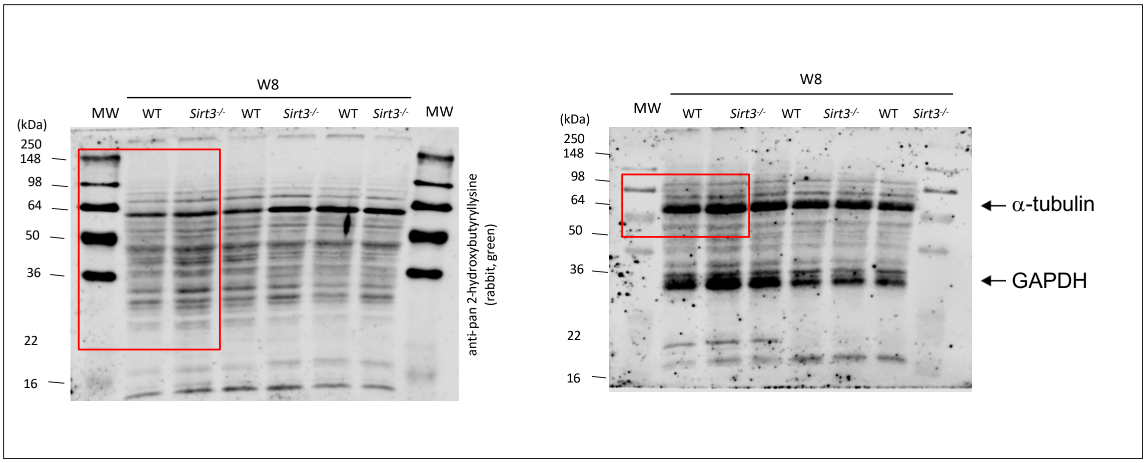

B

PFK Khib in Figure 4B

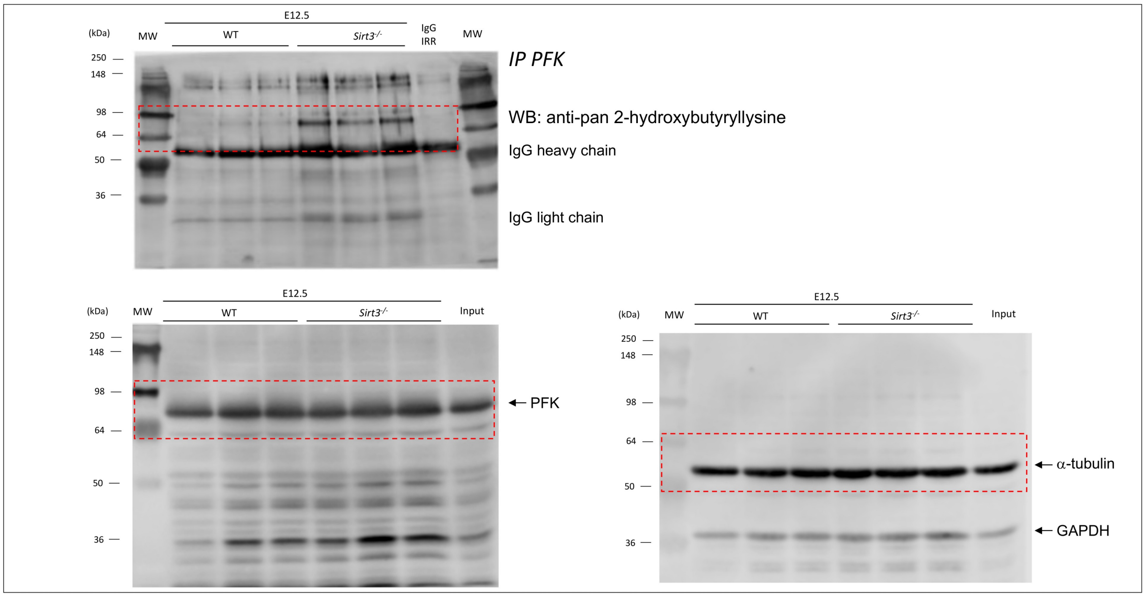

Figure S9. (A) Uncropped gels of lysine 2-hydroxyisobutyrylation (Khib) of total cytoplasmic protein in Fig. 4A and S2A. (**B**) Uncropped Western Blot (WB) gels of Khib in immunoprecipitated (IP) phosphofructokinase (PFK) in Fig. 4B. Normal rabbit IgG was used as an irrelevant isotype control (IRR IgG). Input identifies the basal expression of PFK. Glyceraldehyde-3-phosphate dehydrogenase (GAPDH) or α–tubulin were used as sample loading control for total extracts. Molecular weight (MW) are reported for each gel and expressed in kilo Dalton (kDa). WT, wild type; *Sirt3-/-*, *Sirt3* knockout mice; E12.5, embryonic day 12.5; W8, 8 weeks. Dotted red boxes indicates the portions of gel shown in the main figures of the manuscript.

SOD2 protein expression and SOD2 acetylation in Supplementary Figure 2B

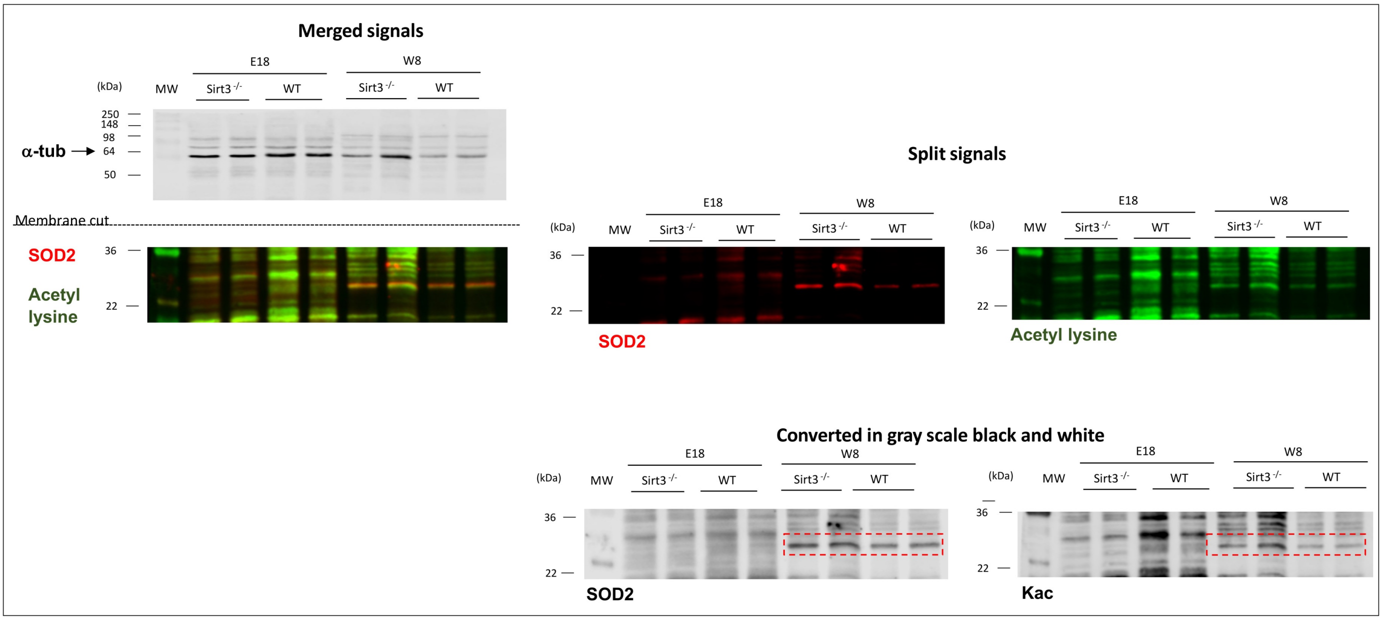

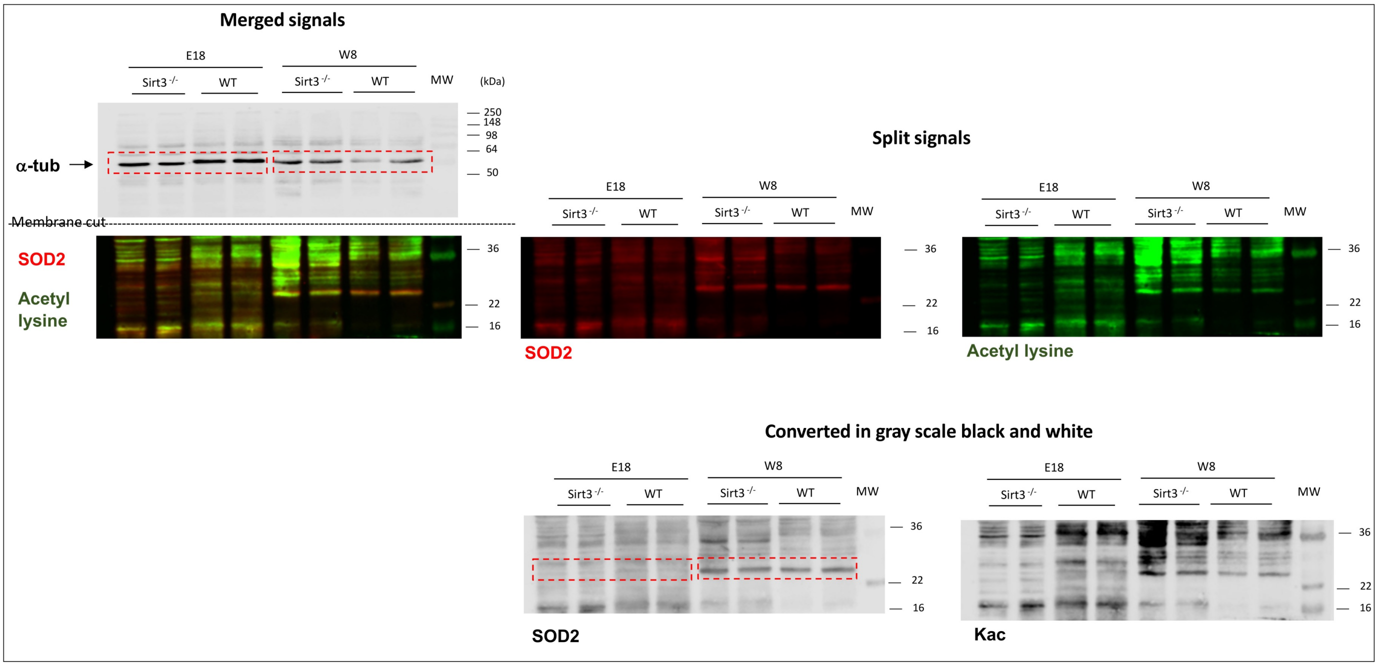

Figure S10. Uncropped gels of the superoxide dismutase 2 (SOD2) protein expression and acetylation (Kac) in Fig. S2B. α-tubulin (α-tub) was used as sample loading control for total/cytoplasmic or mitochondrial extracts, respectively. Molecular weight (MW) are reported for each gel and expressed in kilo Dalton (kDa). WT, wild type; *Sirt3*$^{-/-}$, *Sirt3* knockout mice; E18, embryonic day 18; W8, 8 weeks. Dotted red boxes indicates the portions of gel shown in the main figures of the manuscript.

A

Evaluation of OPA1 acetylation in Supplementary Figure 2C

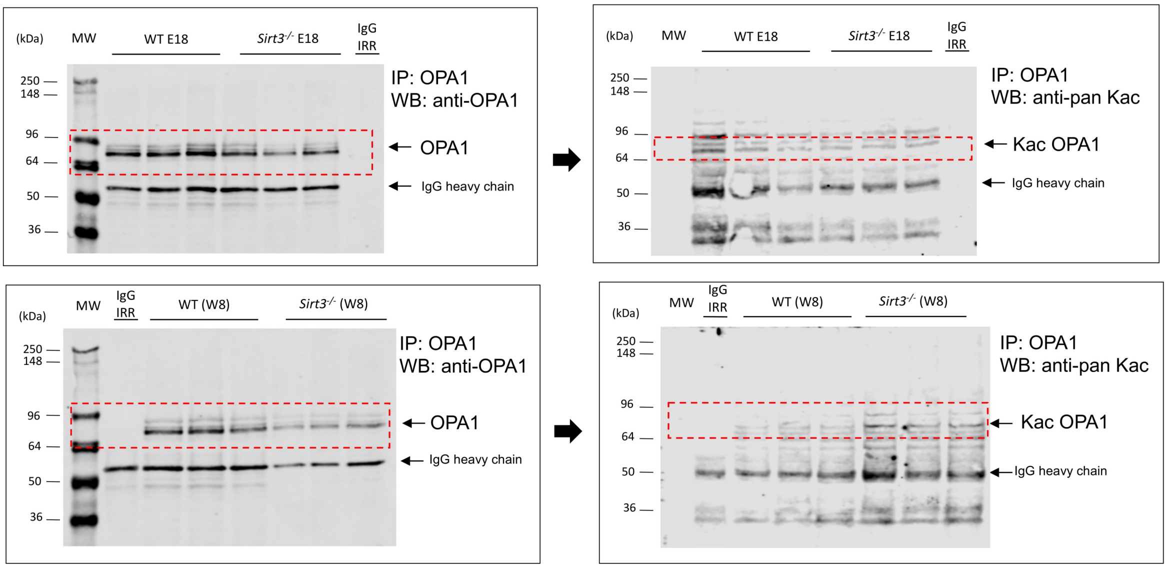

B

PFK Khib in Supplementary Figure 6

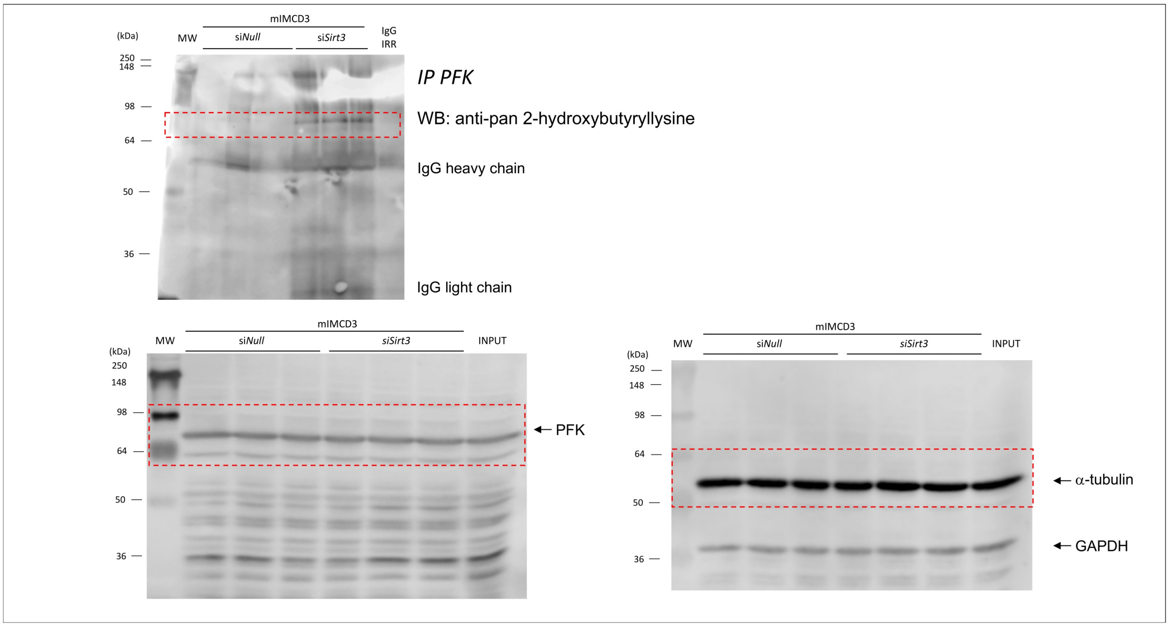

Figure S11. (A) Uncropped gels of lysine acetylation (Kac) of optic atrophy 1 (OPA1) in Fig. S2C. (**B**) Uncropped Western Blot (WB) gels of lysine 2-hydroxyisobutyrylation (Khib) in immunoprecipitated (IP) phosphofructokinase (PFK) in Fig. S6. Normal rabbit IgG was used as an irrelevant isotype control (IRR IgG). Input identifies the basal expression of PFK. Glyceraldehyde-3-phosphate dehydrogenase (GAPDH) or α–tubulin were used as sample loading control for total extracts. Molecular weight (MW) are reported for each gel and expressed in kilo Dalton (kDa). WT, wild type; *Sirt3*$^{-/-}$, *Sirt3* knockout mice; E12.5, embryonic day 12.5; W8, 8 weeks. Dotted red boxes indicates the portions of gel shown in the main figures of the manuscript, mIMCD3, mouse inner medullary collecting duct cells.
